# Single-cell transcriptomics reveals epithelial-stromal crosstalk underlying fibrotic remodeling in frontal fibrosing alopecia

**DOI:** 10.1016/j.isci.2026.116854

**Published:** 2026-07-18

**Authors:** Viviana Dávila-Flores, Jesús Gay-Mimbrera, Carmen Mochón-Jiménez, Irene Rivera-Ruiz, Juan de Luque-Fernández, Pedro J. Gómez-Arias, Beatriz Isla-Tejera, Benjamin Ungar, Benjamin D. Hu, Helen He, Fernando Leiva-Cepas, Emma Guttman-Yassky, Juan Ruano

**Affiliations:** 1 Department of Pathology, Reina Sofía University Hospital, 14004 Córdoba, Spain; 2 Inflammatory Immune-Mediated Chronic Skin Diseases Laboratory, IMIBIC/University of Córdoba, 14004 Córdoba, Spain; 3 Department of Dermatology, Reina Sofía University Hospital, 14004 Córdoba, Spain; 4 Department of Dermatology, Icahn School of Medicine at Mount Sinai, New York, NY, USA; 5 Department of Pharmacy, Reina Sofía University Hospital, 14004 Córdoba, Spain; 6 School of Medicine, University of Cordoba, 14004 Cordoba, Spain

**Keywords:** frontal fibrosing alopecia, single-cell RNA sequencing, pilosebaceous unit, epithelial stress, pseudotime, transcriptional regulation, interferon signaling, circadian disruption, fibroblast activation, fibrosis

## Abstract

Frontal fibrosing alopecia (FFA) is a primary cicatricial alopecia characterized by progressive perifollicular fibrosis and irreversible hair loss. We applied single-cell RNA sequencing to lesional scalp skin from patients with FFA and matched controls to define cellular states, lineage trajectories, and regulatory programs within the pilosebaceous unit. Analysis of 38,984 cells revealed preservation of major epithelial, stromal, and immune populations despite substantial transcriptional remodeling. Epithelial cells exhibited compartment-specific stress-associated reprogramming across follicular and interfollicular domains, progressing along directional trajectories enriched for interferon-STAT/IRF and Th1-related regulatory networks. In contrast, fibroblasts showed limited trajectory remodeling and instead displayed a conserved activation program driven by shared transcriptional regulators. These findings support a model of asymmetric epithelial-stromal dynamics in FFA, suggesting that epithelial stress-associated reprogramming may contribute to stromal activation and fibrotic remodeling and highlighting therapeutic opportunities beyond immune-targeted interventions.

## Highlights

• Single-cell transcriptomics reveals divergent epithelial and stromal responses in FFA
• Immune activation is dominated by IFN-driven T cell responses
• Epithelial cells undergo stress-associated regulatory reprogramming
• Fibroblasts activate epithelial-induced profibrotic programs without fate rewiring

## Graphical abstract

**Figure:**
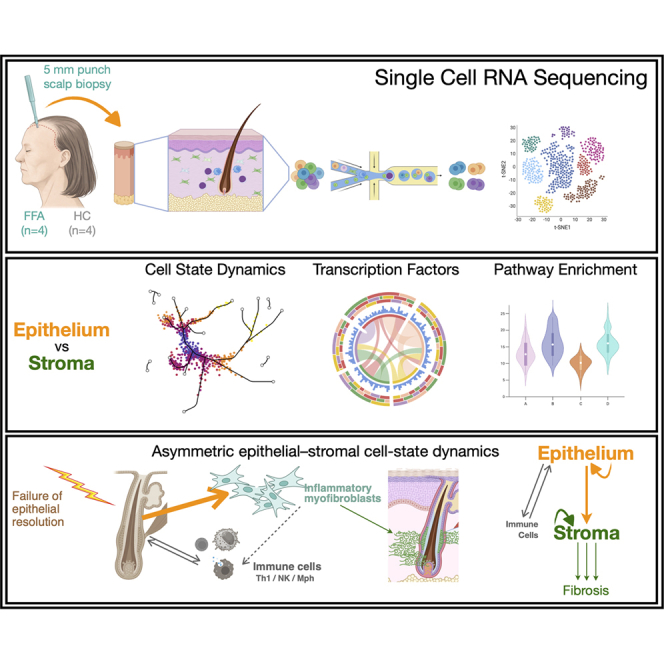

## Teaser

Cell biology; Transcriptomics

## Introduction

Frontal fibrosing alopecia (FFA) is a primary lymphocytic cicatricial alopecia (PLCA) characterized by progressive frontotemporal hairline recession, frequent eyebrow involvement, and irreversible follicular loss.^1^ Despite increasing clinical recognition and incidence, the biological mechanisms sustaining disease persistence and slow progression remain incompletely understood.^2^ Although genetic susceptibility, hormonal factors, and environmental exposures have been implicated, including associations with leave-on facial products and sunscreens as well as disruption of the hair follicle epithelial stem cell niche described in prior studies, no unifying pathogenic framework has yet emerged.^3^^,^^4^^,^^5^^,^^6^^,^^7^^,^^8^^,^^9^^,^^10^^,^^11^^,^^12^^,^^13^^,^^14^

Clinically, FFA follows a slow and often unpredictable course, with gradual follicular attrition often occurring in the absence of marked clinical inflammation.^15^^,^^16^ This indolent behavior, together with the lack of reliable prognostic biomarkers, complicates patient stratification, disease monitoring, and therapeutic decision making.

Current pathogenic models have largely focused on immune-mediated mechanisms, particularly early collapse of hair follicle immune privilege driven by Th1/IFN-γ-skewed cytotoxic responses targeting the upper follicle and bulge stem cell niche.^17^^,^^18^^,^^19^ While supported by histopathological observations and bulk transcriptomic analyses, this immune-centric framework does not fully explain key features of FFA, including the relative preservation of follicular architecture within clinically active, non-end-stage regions; gradual progression; and heterogeneous and variably reported responses to immunosuppressive therapies. Complementing immune-centric models, epithelial-mesenchymal transition (EMT) has been proposed as a contributing mechanism in scarring alopecias, linking epithelial stress responses to fibrotic remodeling and representing a potential therapeutic target.^20^ However, the extent to which EMT-related processes contribute to FFA pathogenesis and how they integrate with epithelial-stromal interactions remains incompletely understood. Moreover, bulk approaches cannot distinguish whether disease-associated transcriptional signatures reflect changes in cellular composition or functional reprogramming within preserved lineages, nor can they resolve the contribution of epithelial and stromal compartments to fibrotic remodeling.

The pilosebaceous unit is a highly organized epithelial structure embedded within a specialized stromal and immune microenvironment, whose homeostasis depends on coordinated epithelial-stromal-immune interactions. Disruption of these relationships, in addition to immune activation, may, therefore, contribute to disease persistence. However, the dynamic cellular programs governing epithelial-stromal coupling in FFA remain poorly defined.

Single-cell transcriptomic technologies enable molecular interrogation at cellular resolution, overcoming the limitations of bulk tissue analyses.^21^^,^^22^^,^^23^ These approaches resolve lineage relationships, functional states, and cell-type-specific responses within complex skin tissues.^24^^,^^25^ Recent single-cell studies in inflammatory and fibrotic skin diseases have demonstrated that epithelial and stromal compartments actively shape disease trajectories through stress responses, altered differentiation programs, and maladaptive intercellular communication.^19^^,^^26^^,^^27^ In contrast, in FFA, the dynamic behavior of individual cellular compartments and the regulatory mechanisms governing their interactions remain largely unexplored.^28^

In particular, it is unknown whether disease-associated epithelial and fibroblast states in FFA represent static activation phenotypes or directed, trajectory-based processes of cellular reprogramming. The transcription factor (TF) networks controlling these processes and whether they differ fundamentally between epithelial and stromal lineages, have not been systematically investigated. Distinguishing regulatory programs potentially associated with disease initiation from downstream effector responses may help to better understand progressive, irreversible fibrosis in FFA.

In this study, we apply single-cell RNA sequencing combined with lineage-specific pseudotime inference and TF activity analysis to lesional scalp skin from patients with FFA and matched controls. By reconstructing dynamic trajectories within epithelial and fibroblast compartments, we define how these lineages diverge in their regulatory responses to disease. This compartment-resolved approach supports a model in which epithelial stress-associated reprogramming may contribute to stromal activation and fibrotic remodeling, whereas fibroblasts respond predominantly through amplification of shared inflammatory and profibrotic activation programs rather than overt lineage rewiring. Together, these findings provide a compartment-resolved regulatory framework for FFA pathogenesis that complements immune-centric models and highlights epithelial-stromal interactions as potential contributors to disease persistence and fibrotic remodeling.

## Results

### Preserved cellular architecture with disease-associated redistribution of functional states

Single-cell transcriptomic profiling identified the major epithelial, stromal, immune, and vascular compartments of frontal scalp skin, revealing a largely preserved global cellular architecture in FFA compared with control samples, despite pronounced disease-associated transcriptional reprogramming. All major scalp lineages were represented in both conditions, with no evidence for the emergence or loss of entire cell types (Figure S2). A per-sample composition audit further confirmed that major compartments remained represented even in lower-cell-count controls (CON266 and CON267), with variability mainly affecting low-frequency populations rather than indicating systematic compartment loss (Figure S5). Subsequent analyses, therefore, focused on epithelial and fibroblast compartments, which represent major cellular compartments implicated in FFA pathogenesis.

**Figure 1:**
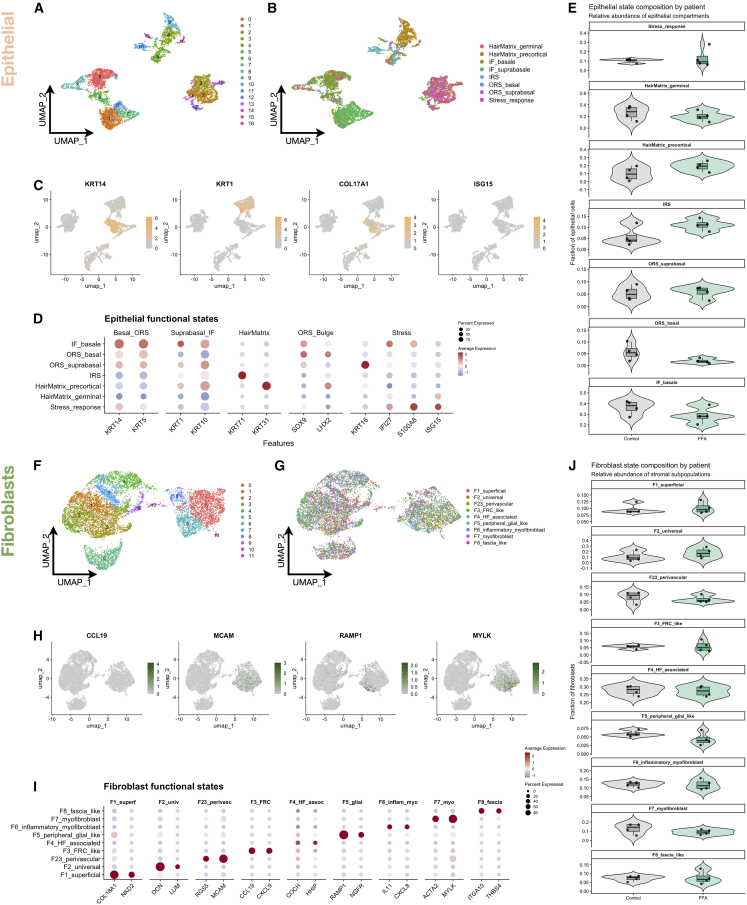
Epithelial and stromal cell-state heterogeneity defines disease-associated compositional remodeling in frontal fibrosing alopecia (A) UMAP of epithelial cells showing unsupervised epithelial subclusters. (B) UMAP colored by annotated epithelial cell states. (C) Feature plots of representative epithelial markers (KRT14, KRT1, COL17A1, and ISG15) used for state annotation. (D) Dot plot summarizing canonical marker expression across epithelial states; dot size indicates the percentage of expressing cells and color indicates average scaled expression. (E) Relative abundance of epithelial states in control and FFA samples; violin plots show patient-level distributions with embedded boxplots indicating the median and interquartile range. (F) UMAP of stromal cells showing unsupervised stromal subclusters. (G) UMAP colored by annotated stromal cell states. (H) Feature plots of representative stromal markers (CCL19, MCAM, RAMP1, and MYLK). (I) Dot plot summarizing canonical marker expression across stromal states. (J) Relative abundance of stromal states in control and FFA samples. Violin plots show patient-level distributions with embedded boxplots indicating the median and interquartile range. Where indicated, statistical significance was assessed through two-sided Wilcoxon rank-sum tests with Benjamini-Hochberg correction (∗*p* < 0.05, ∗∗*p* < 0.01, ∗∗∗*p* < 0.001; n.s., not significant). See also Figures S2–S5; Tables S1 and S2.

Despite preservation of lineage architecture, marked disease-associated differences emerged in the distribution and organization of functional cellular states. Unsupervised subclustering within epithelial and fibroblast compartments resolved multiple transcriptionally distinct states whose relative abundance differed between FFA and control samples, indicating state-level rather than lineage-level remodeling (Figures 1A–1J).

Within the epithelial compartment, keratinocytes segregated into basal, suprabasal, outer root sheath (ORS), inner root sheath (IRS), hair matrix-associated, and stress-response states (Figures 1A–1D; Table 2). All epithelial states were present in both conditions, supporting preservation of epithelial lineage identity. However, FFA samples exhibited a consistent relative enrichment of stress-associated and suprabasal inflammatory states, accompanied by proportional reductions in basal and ORS-associated populations (Figure 1E).

**Table 2:** Definition and functional annotation of epithelial and fibroblast cell state**s**

Compartment	State name	Key marker genes	Functional annotation
Epithelial	IF_basale	KRT14, KRT5, IFI27, and ISG15	basal keratinocytes with interferon-responsive and inflammatory transcriptional profile
	ORS_basal	KRT14, KRT5, and KRT17	basal outer root sheath keratinocytes with progenitor-like features
	ORS_suprabasal	KRT1 and KRT10	differentiating suprabasal outer root sheath keratinocytes
	IRS	KRT71 and KRT73	inner root sheath keratinocytes involved in hair shaft support
	HairMatrix_proximal	SOX9 and LHX2	proliferative hair matrix progenitors with stem-like transcriptional programs
	HairMatrix_germinal	KRT71 and KRT31	germinative hair matrix cells associated with hair shaft formation
	Stress_response	KRT16, IFI27, and S100A8	stress-activated keratinocytes with injury-associated signatures
Fibroblast	F1_superficial	COL1A1, DCN, and LUM	superficial dermal fibroblasts with extracellular matrix homeostasis functions
	F2_universal	COL1A1 and DCN	core fibroblast population shared across dermal regions
	F23_perivascular	RARB and ZHX1	perivascular fibroblasts associated with vascular niches
	F3_FRC_like	CCL19, CXCL9, and ADAMDEC1	fibroblastic reticular-like cells with immune-interacting properties
	F4_HF_associated	FOXN1 and GRHL3	hair-follicle-associated fibroblasts linked to epithelial-mesenchymal crosstalk
	F5_peripheral glial-like	S100B and SOX10	peripheral glial-like stromal cells with neural-associated features
	F6_inflammatory_myofibroblast	CXCL8, IL11, and CXCL5	inflammatory myofibroblasts with chemokine-driven immune recruitment
	F7_myofibroblast	ACTA2 and TAGLN	contractile myofibroblasts linked to tissue remodeling and fibrosis
	F8_fascia_like	ACAN, ITGA10, and THBS4	fascia-like fibroblasts associated with deep stromal architecture

**Figure 2:**
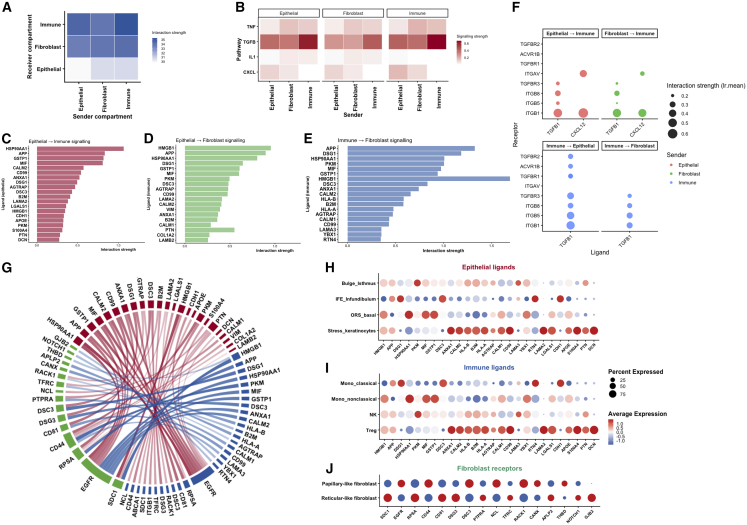
Compartment-level epithelial-stromal-immune communication network in frontal fibrosing alopecia (A) Heatmap summarizing the inferred strength of cell-cell communication between major tissue compartments. (B) Heatmap showing pathway-level signaling activity across epithelial, fibroblast, and immune sender-receiver pairs. (C) Dot plot of representative ligand-receptor interactions linking epithelial, stromal, and immune compartments. Dot size represents inferred interaction strength and color denotes the sender compartment. (D–F) Ranked ligand contributions to the major epithelial-to-immune, epithelial-to-fibroblast, and immune-to-fibroblast signaling axes. (G) Circos plot summarizing dominant ligand-receptor interactions across tissue compartments. (H–J) Dot plots showing ligand expression across epithelial (H) and immune (I) subpopulations and receptor expression across fibroblast subsets (J). Dot size indicates the percentage of expressing cells and color indicates average scaled expression. See also Figure S8.

Cellular states were defined on the basis of unsupervised clustering, marker gene expression, and functional signatures. The table provides a reference framework linking transcriptionally defined cell states shown in Figure 1 with pseudotime trajectory analyses presented in Figure 2, facilitating interpretation for readers not specialized in single-cell transcriptomics.

The broader distribution of canonical epithelial markers (e.g., KRT14 and KRT15) across uniform manifold approximation and projection (UMAP) space likely reflects a combination of biological overlap between closely related epithelial states and technical factors, such as ambient RNA contamination or low-level doublets. Importantly, our analyses rely on integrated transcriptional profiles rather than single-gene expression, minimizing the impact of such effects.

Fibroblasts similarly displayed marked heterogeneity, resolving into superficial, universal, perivascular, fascia-like, peripheral glial-like stromal cells, inflammatory, and myofibroblast-like states (Figures 1F–1I; Table 2). In FFA, inflammatory myofibroblasts and ACTA2^+^ myofibroblast states were selectively expanded, whereas quiescent fibroblast populations were proportionally reduced (Figure 1J).

Together, these findings suggest that FFA is characterized not by cellular replacement or lineage collapse but by disease-associated redistribution across pre-existing epithelial and stromal functional states.

### Orthogonal histopathological support for epithelial niche alteration and perifollicular stromal remodeling in FFA

To provide orthogonal histopathological support for the transcriptomic findings, we performed immunohistochemical and histological analyses of FFA and control scalp sections (Figure S8). FFA samples showed reduced CK15 staining within bulge-associated epithelial regions, together with increased perifollicular SDF1/CXCL12 and α-SMA expression, consistent with stromal signaling activation and myofibroblast-associated remodeling. Masson trichrome staining further demonstrated increased perifollicular collagen deposition in FFA compared with control scalp. Quantitative analyses supported these spatially resolved alterations across epithelial and stromal compartments.

### Disease-specific epithelial-stromal-immune communication networks in FFA

To determine whether these compartmental changes were accompanied by altered intercellular signaling, we reconstructed cell-cell communication networks across epithelial, stromal and immune compartments. At the global level, FFA showed a marked reorganization of compartment-to-compartment interactions, with strong bidirectional communication centered on epithelial, fibroblast and immune populations (Figure 2A). Pathway-level analysis further indicated that this network was dominated by *TNF*-, *TGFB*-, *IL1*-, and *CXCL*-related signaling, with *TGFB*-related activity particularly prominent across compartments (Figure 2B). Examination of representative ligand-receptor pairs highlighted epithelial-to-immune and fibroblast-to-immune signaling through *TGFB1*/*TGFBR* and *CXCL12*/integrin-associated axes, together with reciprocal immune-to-epithelial and immune-to-fibroblast inputs (Figure 2C).

At higher resolution, epithelial cells emerged as major inferred signal senders toward both immune and fibroblast compartments, whereas immune-derived signals were especially prominent toward fibroblasts (Figures 2D–2F). The dominant epithelial ligands included stress- and adhesion-associated molecules such as APP, HSP90AA1, DSG1, GSTP1, MIF, and ANXA1, while immune-derived communication was enriched for inflammatory and antigen-associated mediators including APP, HSP90AA1, MIF, HLA genes, and B2M (Figures 2D–2F). Chord-diagram visualization further showed convergence of epithelial and immune ligand programs onto fibroblast receptor modules, consistent with fibroblasts functioning as major signal integrators in the FFA microenvironment (Figure 2G).

Subpopulation-level mapping refined this architecture by showing that epithelial stress keratinocytes, ORS-basal cells, and bulge/isthmus populations contributed distinct ligand programs, while Treg, natural killer (NK), and monocyte/macrophage populations provided complementary immune-derived signals (Figures 2H and 2I). On the receiving side, fibroblast subsets, including papillary-like and reticular-like fibroblasts, expressed receptor repertoires compatible with epithelial- and immune-derived inputs (Figure 2J). Together, these data support a disease-specific signaling configuration in FFA, in which epithelial stress states may function as major signaling hubs associated with stromal remodeling and immune activation.

### Subpopulation-level communication identifies stressed epithelial and myofibroblast hubs in FFA

**Figure 3:**
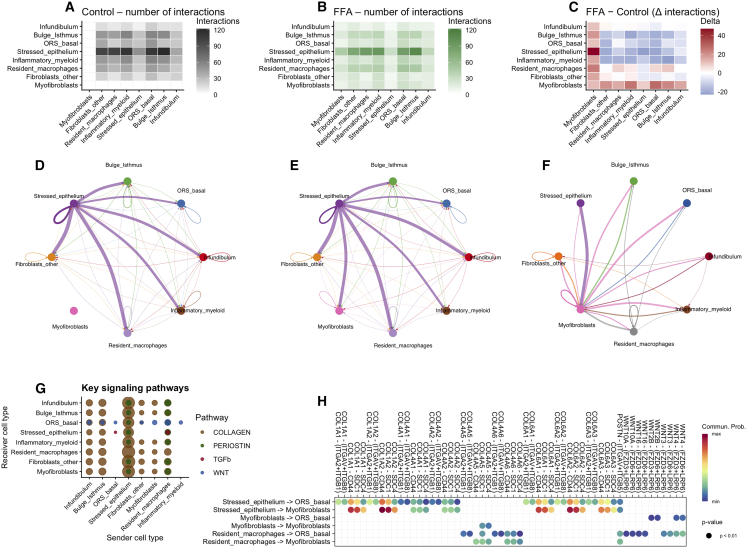
Disease-associated rewiring of epithelial-stromal-immune communication at single-cell resolution (A and B) Heatmaps showing the number of inferred interactions among epithelial, stromal, and immune subpopulations in control (A) and FFA (B). (C) Differential interaction heatmap (FFA-control). Positive values indicate interaction gain in FFA. (D–F) Circle plots summarizing communication networks in control (D), FFA (E), and differential (F) conditions. Node size reflects the contribution of each cell state and edge width reflects interaction strength. (G) Dot plot summarizing major signaling pathways contributing to disease-associated communication. (H–J) Dot plots showing ligand expression across epithelial (H) and immune (I) populations and receptor expression across fibroblast and myofibroblast populations (J). Dot size indicates the percentage of expressing cells, and color indicates average scaled expression.

To refine the compartment-level communication patterns described earlier, we next resolved the FFA signaling network at subpopulation level. This analysis showed that the global epithelial-stromal-immune architecture was not uniformly distributed across all cell states, but instead concentrated in a restricted set of epithelial, mesenchymal, and myeloid subpopulations (Figures 3A–3F).

Among epithelial populations, Stressed_epithelium emerged as a prominent signaling hub, showing the strongest increase in interaction number and connectivity in FFA compared with control (Figures 3B, 3C, 3E, and 3F). By contrast, more homeostatic follicular epithelial populations, including Bulge_Isthmus and ORS_basal, displayed more limited or selective changes, indicating that disease-associated communication rewiring is concentrated predominantly within stressed epithelial states rather than uniformly distributed across epithelial compartments (Figures 3C–3F).

Within the stromal compartment, myofibroblasts and Fibroblasts_other showed enhanced connectivity and participated in many of the interactions gained in FFA, consistent with their role as major stromal responders to epithelial stress (Figures 3C–3F). Immune contributions were largely concentrated in Inflammatory_myeloid and Resident_macrophages, which formed part of the expanded communication network linking stressed epithelium to activated mesenchymal populations (Figures 3A–3F).

Pathway analysis revealed that this disease-specific rewiring was dominated by COLLAGEN, PERIOSTIN, *TGFb*, and WNT signaling, particularly in interactions involving Stressed_epithelium, ORS_basal, fibroblasts, and myofibroblasts (Figure 3G). At the ligand-receptor level, the most prominent FFA-enriched interactions included collagen-integrin, periostin-associated, *TGFB*, and *WNT*/*FZD* axes, supporting a model in which stressed epithelial cells may engage stromal receptor programs associated with matrix remodeling and profibrotic niche conversion (Figure 3H).

### Directional epithelial pseudotime trajectories toward stress-associated terminal states

**Figure 4:**
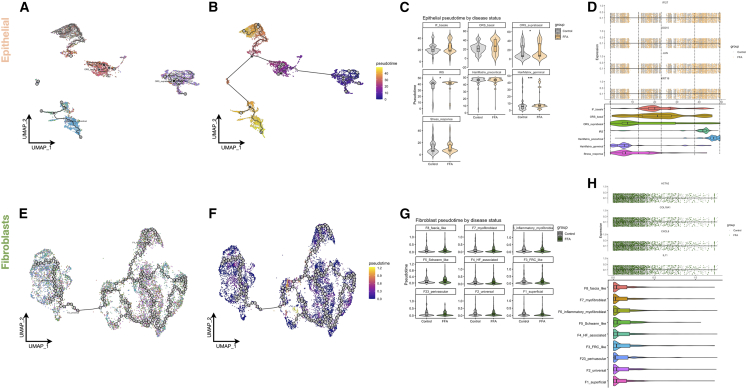
Distinct epithelial and stromal pseudotime trajectories characterize frontal fibrosing alopecia (A and B) Epithelial trajectory inference and pseudotime projection. (C) Distribution of epithelial pseudotime according to disease status across epithelial states. (D) Representative epithelial gene expression dynamics along pseudotime. (E and F) Stromal trajectory inference and pseudotime projection. (G) Distribution of stromal pseudotime according to disease status across stromal states. (H) Representative stromal gene expression dynamics along pseudotime. Violin plots show patient-level distributions with embedded boxplots indicating the median and interquartile range. Where indicated, statistical significance was assessed through two-sided Wilcoxon rank-sum tests with Benjamini-Hochberg correction (∗*p* < 0.05, ∗∗*p* < 0.01, ∗∗∗*p* < 0.001; n.s., not significant).

To investigate whether state redistribution reflected dynamic cellular progression, we reconstructed lineage-specific pseudotime trajectories within the epithelial compartment. Pseudotime inference identified coherent directional trajectories extending from basal and ORS-associated states toward suprabasal, matrix-associated, and stress-response terminal states (Figures 4A–4D). Condition-stratified projections showed that global trajectory topology was largely preserved between control and FFA, with disease effects primarily reflected as state-specific shifts in pseudotime distributions rather than uniform trajectory-wide displacement (Figures 4B and 4C). Accordingly, significant differences were observed in selected epithelial states (including Stress_response, IRS, and HairMatrix_germinal), whereas other states (including IF_basale, ORS_basal, ORS_suprabasal, and HairMatrix_precortical) showed no significant shift (Figure 4C). The same overall structure was observed across samples, supporting the overall consistency of the inferred trajectory structure across samples.

Gene expression dynamics along epithelial pseudotime revealed progressive induction of interferon-, injury- and stress-associated genes, including *IFI27*, *ISG15*, *JUN* and *KRT16*, together with attenuation of epithelial homeostasis-associated programs (Figure 4D). These coordinated changes may reflect epithelial reprogramming in FFA, with branch-dependent stress-associated remodeling rather than a uniform increase in pseudotime across all epithelial states.

To evaluate whether EMT contributes to epithelial remodeling in FFA, we examined canonical EMT markers and a hallmark EMT module score across epithelial states (Figure S6). Core EMT transcription factors (*SNAI1*, *SNAI2*, *ZEB1*, *ZEB2*, and *TWIST1*) showed low and heterogeneous expression, without a coherent pan-epithelial activation pattern. In contrast, epithelial identity markers (*CDH1*, *EPCAM*, and keratins) remained broadly represented across epithelial states. EMT module scores did not show a strong global shift between control and FFA but varied by epithelial state, with relatively higher values in stress/suprabasal and hair-matrix-associated states. Overall, these data support state-restricted EMT-like features rather than a canonical, global EMT program.

At cell level, EMT module score differences were detected in selected epithelial states (Figure S6). However, when analyses were aggregated at patient level (median score per patient and state), these differences were not statistically significant after multiple-testing correction, consistent with limited patient-level power (Figure S7).

### Fibroblast activation within a conserved pseudotime framework

We next examined whether fibroblasts underwent analogous subtle alterations within an overall conserved trajectory framework. In contrast to the epithelial compartment, fibroblast pseudotime showed a comparatively constrained dynamic range and broad overlap between disease groups, with preservation of the overall trajectory topology in FFA and control samples (Figures 4E–4H). Although FFA fibroblasts displayed modest shifts toward later pseudotime positions in subsets associated with inflammatory, myofibroblast-like, or activated stromal features, these changes occurred without clear evidence of major branching or lineage redirection (Figure 4G).

Instead, gene expression dynamics along fibroblast pseudotime showed gradual changes in extracellular matrix, contractility, and inflammatory mediators, including *ACTA2*, *COL1A1*, *CXCL8*, and *IL11* (Figure 4H), supporting the interpretation of a diffuse activation continuum without clear trajectory bifurcation. These findings suggest that fibroblasts in FFA primarily engage a conserved activation continuum, amplifying fibrotic and inflammatory programs without undergoing the stronger activation-associated progression observed in epithelial cells.

### Distinct epithelial and fibroblast regulatory programs underlie FFA-associated state transitions

**Figure 5:**
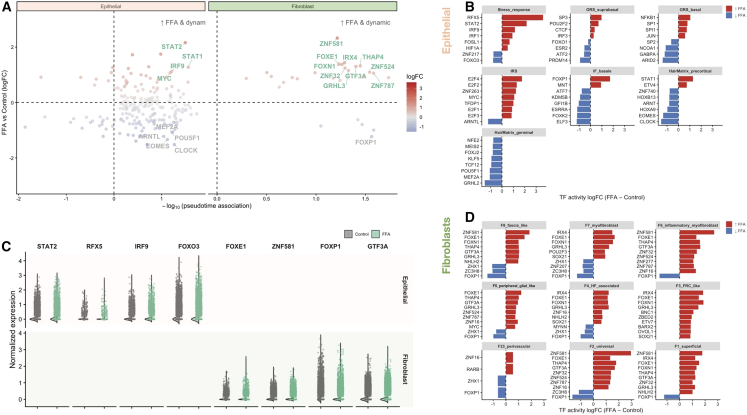
Transcription factor programs associated with disease progression and pseudotime remodeling in frontal fibrosing alopecia (A) Scatterplot integrating transcription factor activity associated with pseudotime progression and differential activity in FFA relative to controls. (B) Differential transcription factor activity across epithelial states. (C) Expression distributions of representative transcription factors in epithelial and fibroblast compartments from control and FFA samples. (D) Differential transcription factor activity across fibroblast states. Red indicates higher inferred activity in FFA and blue indicates lower inferred activity relative to controls.

To identify transcriptional regulators associated with the disease-linked dynamic changes described earlier, we next examined TF activity in epithelial and fibroblast compartments. Joint analysis of pseudotime association and differential activity between FFA and control highlighted compartment-specific regulatory programs, with epithelial cells showing enrichment of interferon- and stress-associated factors such as *STAT2*, *STAT1*, *IRF9*, *IRF7*, and *RFX5*, whereas fibroblasts were characterized by activation of a distinct set of regulators, including *ZNF581*, *FOXE1*, *IRX4*, *THAP4*, *GTF3A*, and *ZNF524* (Figure 5A).

At subpopulation resolution, epithelial stress-response states showed the strongest gain of inflammatory and interferon-related TF activity in FFA, including *RFX5*, *STAT2*, *IRF9*, *IRF1*, and *HIF1A*, while ORS-basal and suprabasal populations displayed more selective regulatory shifts (Figure 5B). In parallel, matrix- and precursor-like epithelial states were characterized by relative depletion of developmental and homeostatic regulators in FFA, consistent with disease-associated remodeling of epithelial identity programs (Figure 5B).

Fibroblast states exhibited a different regulatory architecture. Across fascia-like, myofibroblast-like, inflammatory myofibroblast-like, and HF-associated fibroblast populations, FFA was associated with increased activity of *ZNF581*, *FOXE1*, *FOXN1*, *THAP4*, *GTF3A*, *GRHL3*, and related factors, whereas *FOXP1* showed consistent relative depletion across several fibroblast subsets (Figure 5D). This pattern supports activation of a shared fibroblast-associated regulatory module rather than emergence of entirely distinct lineage trajectories.

Expression-level analysis of representative regulators further supported this compartmental split, with epithelial-biased induction of *STAT2*, *RFX5*, and *IRF9*, and fibroblast-associated enrichment of *FOXE1*, *ZNF581*, *FOXP1*, and *GTF3A* across disease groups (Figure 5C). Together, these results suggest that FFA progression is accompanied by coordinated but compartment-specific regulatory remodeling.

### Compartment-specific pathway enrichment reveals divergent functional consequences of epithelial reprogramming and fibroblast activation

To define the functional programs associated with the compartment-specific regulatory changes observed in FFA, we performed reactome pathway enrichment analysis by using genes linked to pseudotime progression and TF activity in epithelial and fibroblast compartments (Tables 3 and 4).

**Table 3:** Reactome pathway enrichment of epithelial transcriptional programs associated with pseudotime progression and transcription factor activity in FFA

Reactome ID	Pathway description	Gene ratio	Adjusted *p* value	Functional interpretation
R-HSA-877300	interferon gamma signaling	23/632	3.26 × 10^−6^	core IFN-STAT-IRF stress program driving epithelial activation
R-HSA-913531	interferon signaling	39/632	2.34 × 10^−5^	broad interferon-mediated innate immune response
R-HSA-909733	interferon alpha/beta signaling	18/632	2.43 × 10^−5^	type I interferon response linked to epithelial stress
R-HSA-193648	NRAGE signals death through JNK	16/632	2.40 × 10^−5^	stress-induced apoptotic and JNK signaling
R-HSA-204998	cell death signaling via NRAGE, NRIF, and NADE	18/632	2.40 × 10^−5^	damage-associated cell death pathways
R-HSA-73887	death receptor signaling	22/632	1.86 × 10^−3^	extrinsic apoptosis and inflammatory cell death
R-HSA-2990846	SUMOylation	30/632	2.63 × 10^−5^	post-transcriptional and stress-adaptive regulation
R-HSA-3108232	SUMO E3 ligases SUMOylate target proteins	30/632	2.40 × 10^−5^	fine-tuning of transcription factor activity
R-HSA-9012999	RHO GTPase cycle	53/632	2.63 × 10^−5^	cytoskeletal remodeling and epithelial dynamics
R-HSA-6785807	interleukin-4 and interleukin-13 signaling	22/632	2.40 × 10^−5^	Th2-associated epithelial inflammatory signaling
R-HSA-400253	circadian clock	16/632	9.75 × 10^−5^	temporal regulation linked to chronic stress
R-HSA-9006936	signaling by TGFB family members	25/632	1.72 × 10^−4^	crosstalk with fibrotic and stromal pathways

Reactome pathway enrichment analysis of genes associated with epithelial pseudotime dynamics and transcription factor activity in frontal fibrosing alopecia (FFA). Enriched pathways highlight a dominant interferon-driven stress response, apoptosis and damage signaling, post-transcriptional regulation, cytoskeletal remodeling, and Th1/Th2-related epithelial activation.

**Table 4:** Reactome pathway enrichment of fibroblast transcriptional programs associated with pseudotime progression and transcription factor activity in FFA

Reactome ID	Pathway description	Gene ratio	Adjusted *p* value	Functional interpretation
R-HSA-156827	L13a-mediated translational silencing of ceruloplasmin expression	23/434	3.25 × 10^−8^	translational control and post-transcriptional regulation
R-HSA-72613	eukaryotic translation initiation	23/434	5.84 × 10^−8^	global activation of protein synthesis
R-HSA-72737	Cap-dependent translation initiation	23/434	5.84 × 10^−8^	increased mRNA translation efficiency
R-HSA-156902	peptide chain elongation	18/434	1.43 × 10^−6^	sustained protein production
R-HSA-72764	eukaryotic translation termination	18/434	1.96 × 10^−6^	completion and regulation of translational output
R-HSA-8868773	rRNA processing in the nucleus and cytosol	25/434	1.03 × 10^−5^	ribosome biogenesis and biosynthetic capacity
R-HSA-9633012	response of EIF2AK4 (GCN2) to amino acid deficiency	19/434	1.68 × 10^−6^	metabolic stress and nutrient-sensing adaptation
R-HSA-71291	metabolism of amino acids and derivatives	34/434	1.05 × 10^−4^	support of biosynthetic and energetic demands
R-HSA-9711097	cellular response to starvation	22/434	1.10 × 10^−5^	adaptation to chronic metabolic stress
R-HSA-6785807	interleukin-4 and interleukin-13 signaling	19/434	2.87 × 10^−6^	Th2-associated fibroblast activation
R-HSA-3700989	transcriptional regulation by TP53	32/434	3.91 × 10^−4^	cell-cycle checkpoint and stress surveillance
R-HSA-453279	mitotic G1 phase and G1/S transition	19/434	1.99 × 10^−4^	controlled proliferative activity
R-HSA-9010553	regulation of expression of SLITs and ROBOs	21/434	1.37 × 10^−4^	tissue organization and fibroblast-epithelial crosstalk
R-HSA-2990846	SUMOylation	21/434	4.83 × 10^−4^	fine-tuning of transcription factor activity

Reactome pathway enrichment analysis of genes associated with fibroblast pseudotime dynamics and transcription factor activity in frontal fibrosing alopecia (FFA). Enriched pathways indicate activation of translational machinery, proteostasis, metabolic stress responses and controlled cell-cycle regulation, consistent with a functionally activated but transcriptionally stable fibroblast state.

In the epithelial compartment, enrichment analysis revealed a dominant stress- and interferon-associated functional landscape (Table 3). The most significant pathways included interferon gamma signaling, interferon signaling, and interferon alpha/beta signaling, consistent with the STAT- and IRF-centered regulatory programs identified along epithelial pseudotime trajectories. In parallel, enrichment of NRAGE signals death through JNK; cell death signaling via NRAGE, NRIF, and NADE; and death receptor signaling indicated activation of damage-response and apoptosis-related pathways. Additional enrichment of SUMOylation, RHO GTPase cycle, and circadian clock pathways further suggested broad post-transcriptional regulation, cytoskeletal remodeling, and disruption of epithelial homeostatic control. Notably, enrichment of signaling by TGFB family members is consistent with a potential functional link between epithelial stress-associated reprogramming and stromal activation.

By contrast, fibroblast-associated genes showed a markedly different enrichment profile (Table 4). The strongest signals were observed for pathways related to translation, ribosome biogenesis, and proteostasis, including eukaryotic translation initiation, Cap-dependent translation initiation, peptide chain elongation, and rRNA processing in the nucleus and cytosol. Enrichment of response of EIF2AK4 (GCN2) to amino acid deficiency, metabolism of amino acids and derivatives, and cellular response to starvation further pointed to metabolic adaptation and sustained biosynthetic activity. Although inflammatory pathways such as interleukin-4 and interleukin-13 signaling were also detected, they appeared secondary to the dominant translational and metabolic signature. Enrichment of transcriptional regulation by TP53 and mitotic G1 phase and G1/S transition supports a controlled activation state, compatible with fibroblast engagement in tissue remodeling rather than major lineage reprogramming.

Together, these compartment-resolved enrichment profiles indicate that epithelial cells in FFA exhibit stress-associated regulatory reprogramming with strong interferon, damage-response, and profibrotic signaling features, whereas fibroblasts predominantly display a biosynthetic, metabolic, and effector-like activation program in association with epithelial and immune-derived cues.

### Integrated model of epithelial-associated stromal activation in FFA

Collectively, our analyses support a model of asymmetric disease organization in FFA, in which epithelial and stromal compartments make distinct contributions to pathology. Epithelial cells exhibit directional, stress-associated reprogramming accompanied by interferon- and injury-related regulatory activation, whereas fibroblasts respond predominantly through amplification of conserved inflammatory, biosynthetic, and fibrotic effector programs without evidence of major lineage redirection. Within this framework, stressed epithelial states emerge as prominent signaling populations associated with stromal activation and altered immune communication, providing a potential mechanistic framework for persistent tissue remodeling and progressive fibrosis in FFA.

## Discussion

In this study, we delineate the dynamic and regulatory logic governing epithelial and stromal behavior in FFA by integrating pseudotime inference with TF activity at single-cell resolution. Rather than cataloging static compositional changes, this approach captures how disease-associated cellular states are established, stabilized, and maintained within relatively preserved epithelial and stromal lineages in the sampled regions. A key outcome of this framework is the identification of a marked asymmetry in regulatory dynamics between epithelial compartments and fibroblasts, with direct implications for disease persistence and therapeutic refractoriness. Our data support a model of FFA characterized by asymmetric, compartment-specific regulatory dynamics, rather than a consequence of global immune activation or extensive lineage loss.

While the physiological human hair cycle involves highly coordinated and reversible transcriptional reprogramming across epithelial and mesenchymal compartments,^28^ FFA represents a pathological deviation from these programs, characterized by sustained epithelial stress signaling and persistent stromal activation rather than immediate or overt irreversible lineage disruption within the sampled regions. In this context, fibrosis in the sampled regions appears more consistent with persistent stress-associated activation states than with overt aberrant cell fate conversion, but from failure to re-establish tissue resolution mechanisms.

A central finding of this study is the highly ordered, identity-preserving nature of epithelial reprogramming in FFA. Epithelial cells segregate into discrete functional domains that follow aligned, non-intersecting pseudotime trajectories, consistent with directionally constrained progression toward stress-associated terminal states rather than indiscriminate plasticity. This canalized behavior argues against stochastic epithelial activation and instead supports regulated reprogramming operating within preserved structural niches of the pilosebaceous unit. Such organization is consistent with recent spatial and single-cell studies of human skin and hair follicles, which demonstrate tight coupling between epithelial identity, spatial position, and functional state rather than chaotic fate transitions.^28^

These observations reinforce the concept that epithelial cells appear to function as active signaling compartments involved in tissue stress responses rather than passive inflammatory targets. In FFA, epithelial compartments preferentially engage interferon-, STAT/IRF-, and stress-response networks,^29^ consistent with a potential role in coordinating inflammatory and fibrotic responses. This interpretation aligns with accumulating evidence across inflammatory skin disorders identifying keratinocytes as central regulators of immune tone, stromal behavior, and disease chronicity.^21^

Taken together, our results position FFA as a disorder associated with maladaptive functional reprogramming within functionally preserved epithelial and stromal compartments, rather than immediate or widespread lineage loss within the sampled regions, which likely reflect clinically active disease stages rather than fully fibrotic end-stage tissue.

These findings should be interpreted in the context of the sampled tissue. While progressive follicular destruction and lineage loss are well-established features of FFA, our data primarily capture clinically active regions, where functional reprogramming precedes overt structural loss. Accordingly, lineage depletion and follicular dropout may occur at later stages of disease progression and may not be fully represented in the analyzed samples.

### Spatial-functional organization of epithelial reprogramming

Beyond transcriptional dynamics, epithelial and fibroblast compartments diverge markedly in their spatial-functional organization. Epithelial cells segregate into well-defined functional domains, each associated with a coherent and internally ordered pseudotime trajectory. These epithelial “islands” remain structurally separated, yet their trajectories are aligned along a shared directional axis without crossing or chaotic branching, indicating tight architectural constraint rather than unrestricted plasticity.^30^^,^^31^

This ordered organization mirrors observations in human hair follicle systems, where epithelial compartments display coordinated spatial and mechanical dynamics that preserve follicular architecture while permitting directional growth and remodeling.^32^ Within this framework, epithelial trajectories in FFA appear to represent stress-adapted deviations within a conserved organizational scaffold rather than *de novo* fate transitions.

By contrast, fibroblast populations do not segregate into sharply defined territories but are spatially dispersed, despite enrichment for inflammatory and myofibroblast-like states. Pseudotime directionality within fibroblasts is weak and diffuse, consistent with activation without canalized trajectory progression. This pattern supports a model in which fibroblasts amplify shared activation programs in response to epithelial- and immune-associated cues rather than engaging lineage- or fate-directed transitions.

### Temporal and metabolic constraints on epithelial reprogramming

The canalized nature of epithelial pseudotime trajectories suggests that disease-associated reprogramming is constrained by intrinsic temporal and metabolic regulatory systems rather than reflecting unrestricted epithelial plasticity. In skin, circadian clock integrity tightly coordinates epithelial stem cell proliferation, DNA repair, and metabolic homeostasis, while inflammatory states suppress physiological oscillations and promote cumulative stress.^33^

Although *BMAL1* (*ARNTL*) and *CLOCK* are classically viewed as core circadian regulators, they also act as transcriptional timing integrators modulating the amplitude and duration of key stress-related pathways, including NF-κB, MAPK/ERK, TGF-β, and HIF-1α signaling—pathways independently implicated in follicular inflammation, stress adaptation, and fibrotic remodeling.^34^ Accordingly, the reduced activity of *BMAL1* and *CLOCK* observed in FFA epithelial states is unlikely to represent a primary pathogenic trigger but rather a secondary consequence of sustained inflammatory and stress signaling that disrupts circadian gating and temporal control.^35^

The hair follicle itself is a self-organizing, oscillatory mini-organ, coordinated through tightly regulated cycles of proliferation, regression, and quiescence.^36^ Disruption of these intrinsic timing mechanisms has long been linked to hair growth disorders characterized by impaired phase transitions. Within this framework, the aligned and non-intersecting epithelial trajectories observed in FFA are consistent with stress-induced reprogramming that remains confined within a pre-existing oscillatory architecture rather than stochastic epithelial activation. Loss of circadian control may, therefore, act as a disease-amplifying mechanism, stabilizing maladaptive epithelial states and preventing re-entry into homeostatic cycling.^37^

Recent spatial and single-cell analyses of human skin aging further support this model. Senescence-associated programs accumulate in a compartment-specific manner, preferentially affecting epithelial domains involved in stress sensing and regeneration while preserving lineage identity.^38^ Importantly, these states are defined by persistent inflammatory and metabolic signaling rather than fate conversion, closely paralleling the epithelial stress states observed in FFA. This framework is compatible with the strong female and postmenopausal predominance of FFA, positioning aging-related loss of epithelial resilience as a permissive amplifier rather than a primary disease trigger.

### Fibroblast activation as a downstream, non-reprogrammed response

In contrast to epithelial cells, fibroblast dynamics in FFA are characterized by activation without trajectory restructuring. Fibroblast populations displayed diffuse pseudotime distributions without evidence of trajectory restructuring, instead exhibiting a conserved activation response driven by shared transcriptional regulators, consistent with amplification of common inflammatory and biosynthetic programs rather than lineage reprogramming. This argues against fibroblast-intrinsic fate conversion as a primary driver of fibrosis and instead supports a model in which fibroblasts predominantly act as downstream effector populations of epithelial-derived cues, with immune signals acting as modulators rather than obligate intermediates.^39^

At the regulatory level, fibroblast TF activity is dominated by a restricted, shared core—including *FOXE1*, *FOXN1*, ZNF family members, *GTF3A*, and *GRHL3*—with uniform downregulation of *FOXP1*, a known repressor of fibroblast activation. These factors relate to basal transcriptional capacity and tissue organization rather than lineage specification, and their uniformity across fibroblast states supports amplification of conserved activation programs rather than TF-driven reprogramming.^40^

This regulatory simplicity contrasts sharply with the epithelial compartment, where pseudotime progression is governed by state-specific STAT/IRF-centered modules. Together, these findings reveal a marked asymmetry in regulatory logic, with epithelial cells showing more pronounced regulatory remodeling of disease-associated transcriptional programs and fibroblasts functioning as integrative responders.^41^ Epithelial cells emerge as prominent regulatory and signaling populations that undergo canalized stress-driven reprogramming, while fibroblasts act as downstream effectors amplifying conserved fibrotic programs without intrinsic fate rewiring.

Functional pathway enrichment further reinforces this division. Epithelial cells preferentially engage immune-stress signaling networks, whereas fibroblast enrichment is dominated by translational, ribosomal, and metabolic pathways, reflecting execution of biosynthetic and remodeling functions rather than immune-centric reprogramming. This activation mode mirrors single-cell models of wound healing, where fibroblasts transiently adopt inflammatory states before stabilizing into long-lasting, non-interconverting populations.^42^

### Multicellular reprogramming and therapeutic implications

Epithelial-stromal interactions may remain partially sustained independently of overt immune-cell abundance changes, providing a plausible explanation for why immune-targeted therapies alone may be insufficient to halt fibrotic progression. Collectively, these findings provide a biologically coherent framework for the slow, progressive, and treatment-resistant nature of FFA. Directional epithelial reprogramming generates a persistent source of stress and pro-fibrotic cues, while fibroblasts sustain tissue remodeling through conserved activation programs. Importantly, this epithelial-stromal axis appears partially uncoupled from immune cell dynamics, providing a plausible explanation for why immune-targeted therapies alone often fail to halt fibrotic progression.

Beyond mechanistic insights, these findings may also inform therapeutic strategies. The identification of epithelial stress-driven transcriptional reprogramming, particularly involving interferon-associated regulatory networks, suggests that targeting pathways such as JAK/STAT signaling may be beneficial, especially in early or clinically active disease stages. In addition, the conserved activation of fibroblasts highlights the potential relevance of antifibrotic approaches aimed at modulating extracellular matrix remodeling and stromal activation. More broadly, restoring epithelial-stromal homeostasis may represent a promising therapeutic direction.

Our results align with emerging evidence across chronic, non-resolving tissue contexts, where coordinated multicellular reprogramming—rather than primary irreversible lineage loss—underpins persistent disease states.^43^ In this light, FFA can be conceptualized as a disease associated with maladaptive epithelial regulatory programs that may contribute to stromal activation and fibrosis.

Building on this framework, we next considered whether the epithelial stress-associated trajectory shifts observed in FFA could reflect activation of an EMT axis. In our dataset, the EMT-related signal was limited and heterogeneous across epithelial states, without evidence of a coherent pan-epithelial EMT program. Accordingly, these findings are not consistent with a uniform, full epithelial-to-mesenchymal transition as the predominant mechanism in FFA epithelium. Rather, they may reflect partial, context-dependent EMT-like features embedded within broader epithelial stress and differentiation programs. This interpretation is consistent with retained epithelial identity-marker expression and with our trajectory/regulatory analyses, which are more compatible with branch-dependent epithelial remodeling than with global transdifferentiation.

From a translational perspective, our findings support strategies that move beyond broad immunosuppression, instead targeting epithelial stress responses, STAT/IRF-driven reprogramming, or fibroblast activation states to stabilize tissue dynamics and potentially achieve more durable disease control. A key translational challenge will be to determine whether the epithelial stress, interferon, and stromal activation programs identified here can be robustly captured using clinically scalable platforms such as bulk RNA profiling or tape-strip transcriptomics, thereby enabling biomarker development for diagnosis, patient stratification, and therapeutic monitoring. In this regard, recent work showing that tape-strip transcriptomics can resolve disease-dependent accessibility of hair follicle compartments supports its potential as a minimally invasive platform for tracking compartment-specific pathogenic programs in inflammatory alopecias.^44^^,^^45^ More broadly, by resolving directionality and regulatory logic at single-cell resolution, our study moves beyond descriptive atlases and provides a dynamic framework for understanding disease persistence in FFA.

In summary, our study supports a model in which FFA is characterized by asymmetric cellular dynamics across epithelial and stromal compartments. Epithelial cells undergo directional, stress-associated reprogramming and appear to occupy central positions within stress-associated signaling and regulatory networks, whereas fibroblasts respond predominantly by amplifying conserved inflammatory, biosynthetic, and fibrotic programs without evidence of major lineage redirection. By integrating pseudotime trajectories, TF activity, and compartment-specific functional profiling, our findings support epithelial-stromal disequilibrium as a key feature of disease persistence in FFA and a plausible barrier to durable therapeutic control.

### Limitations of the study

Despite the insights provided by this study, several limitations should be acknowledged.

(1) Sample size: single-cell RNA sequencing was performed on a limited number of clinically active FFA and control scalp biopsies, which may restrict the detection of rare cellular populations and limit generalizability.
(2) Cross-sectional design: the study captures a single time point and, therefore, cannot directly assess temporal disease progression or establish causal relationships between epithelial and stromal changes.
(3) Computational inference: pseudotime trajectories, transcription^46^ factor activities, and cell-cell communication networks were inferred computationally and require experimental validation.
(4) Disease stage representation: analyzed samples were obtained from^47^ clinically active regions rather than fully fibrotic end-stage lesions and may, therefore, not capture molecular events associated with advanced follicular destruction.
(5) Spatial validation: cell-cell interactions were reconstructed^48^ from dissociated single-cell transcriptomes and do not directly demonstrate spatial organization of epithelial-stromal signaling niches within lesional^27^ tissue.

## Resource availability

### Lead contact

Further information and requests for resources, data, or materials should be directed to and will be fulfilled by the lead contact, Juan Ruano (email: juanruanoruiz@mac.com).

### Materials availability

This study did not generate new unique reagents.

### Data and code availability

• The single-cell RNA sequencing data generated during this study have been deposited in the Gene Expression Omnibus (GEO) under accession number GSE314972 and are publicly available.
• This study does not report custom original code.
• All analyses were performed using publicly available software as detailed in the key resources table.
• Additional information is available from the lead contact upon reasonable request.

## Acknowledgments

We thank the patients and the healthy volunteers whose participation made this study possible. We also thank the CABIMER Bioinformatics Core (Sevilla, Spain) and the clinical teams involved in sample acquisition and processing. This work forms part of the PhD thesis of V.D.-F., conducted within the framework of the Official Doctoral Programme in Biomedicine at the University of Córdoba (Spain). The study was supported by the Plan Propio de Investigación of the Instituto Maimónides de Investigación Biomédica de Córdoba (IMIBIC), with award granted to J.G.-M. P.J.G.-A. received additional support from the International Eczema Council 2022 Research Fellowship Program. This research was funded exclusively by public institutions. Partial financial support was provided by Project PI23/01590 (awarded to J.R.), funded by the 10.13039/501100004587Instituto de Salud Carlos III (ISCIII) and co-financed by the 10.13039/501100000780European Union. The funding bodies had no involvement in study design, data collection or analysis, manuscript preparation, or the decision to submit for publication. No financial support was received from pharmaceutical companies. No funding or sponsorship was received for the publication of this article.

## Author contributions

Conceptualization, C.M.-J., I.R.-R., P.J.G.-A., E.G.-Y., and J.R.; methodology, J.d.L.-F. and J.R.; investigation, V.D.-F., J.G.-M., C.M.-J., I.R.-R., and P.J.G.-A.; formal analysis, C.M.-J. and J.d.L.-F.; bioinformatic analysis, J.G.-M., H.H., and B.D.H.; data curation, V.D.-F., J.G.-M., and I.R.-R.; visualization, C.M.-J.; resources, F.L.-C., B.I.-T., B.U., E.G.-Y., and J.R.; writing – original draft, V.D.-F., C.M.-J., and J.R.; writing – review and editing, V.D.-F., J.G.-M., C.M.-J., I.R.-R., J.d.L.-F., P.J.G.-A., B.I.-T., B.U., B.D.H., H.H., F.L.-C., E.G.-Y., and J.R.; funding acquisition, J.R.; supervision, F.L.-C., E.G.-Y., and J.R. V.D.-F. and J.G.-M. contributed equally to this work as co-first authors. E.G.-Y. and J.R. contributed equally to this work as co-senior authors. All authors had full access to the study data, critically reviewed the manuscript, and approved the final version.

## Declaration of interests

The authors declare no competing interests.

## Declaration of generative AI and AI-assisted technologies in the writing process

During the preparation of this work, the authors used ChatGPT (OpenAI) to assist with language editing, manuscript organization, and programming support. All AI-generated suggestions were critically reviewed, verified, and edited by the authors. The authors take full responsibility for the content of this publication.

## STAR★Methods

### Key resources table

**Table:** 

REAGENT or RESOURCE	SOURCE	IDENTIFIER
**Antibodies**		

Anti-CK15 (EPR1614Y)	Abcam	Cat# ab52816
Anti-CXCL12/SDF1	Abcam	Cat# ab9797
Anti-α-SMA (ASM-1)	Leica Biosystems	Cat# PA0943

**Biological samples**		

Human frontal fibrosing alopecia scalp biopsies	Reina Sofía University Hospital	IRB approval JAAK23
Human healthy control scalp biopsies	Reina Sofía University Hospital	IRB approval JAAK23

**Chemicals, peptides, and recombinant proteins**		

Liberase TM	Roche	Cat# 5401119001
Trypsin-EDTA (0.25%)	Gibco	Cat# 25200056
SPRIselect Reagent Kit	Beckman Coulter	Cat# B23318

**Critical commercial assays**		

Chromium Next GEM Single Cell 3′ Kit v3.1 Dual Index	10× Genomics	Cat# PN-1000268∗
Chromium Next GEM Chip G	10× Genomics	Cat# PN-1000120∗
Masson Trichrome Stain Kit	Leica Biosystems	N/A

**Deposited data**		

Single-cell RNA sequencing dataset	GEO	GSE314972

**Software and algorithms**		

Cell Ranger v3.1.0	10× Genomics	https://support.10xgenomics.com
Seurat v4.0	Stuart et al., 2019	https://satijalab.org/seurat
Monocle3 v1.3.1	Cao et al., 2019	https://cole-trapnell-lab.github.io/monocle3
UMAP	Becht et al., 2019	https://github.com/lmcinnes/umap
UCell v1.3.1	Andreatta and Carmona, 2021	https://github.com/carmonalab/UCell
decoupleR v2.6.0	Badia-i-Mompel et al., 2022	https://bioconductor.org/packages/decoupleR
DoRothEA v1.10.0	Garcia-Alonso et al.^49^	https://saezlab.github.io/dorothea
CellChat v1.5.0	Jin et al., 2021	https://github.com/sqjin/CellChat
R v4.3.1	R Foundation	https://www.r-project.org
Python v3.10	Python Software Foundation	https://www.python.org
ImageJ	NIH	https://imagej.nih.gov/ij/

**Other**		

Countess 3 FL Automated Cell Counter	Thermo Fisher Scientific	N/A
Chromium Controller	10× Genomics	N/A
Bioanalyzer/TapeStation	Agilent Technologies	N/A
Falcon 70 μm Cell Strainer	Corning Falcon	Cat# 352350
Falcon 40 μm Cell Strainer	Corning Falcon	Cat# 352340

### Experimental model and study participant details

#### Human tissue samples

This study examined lesional frontal scalp skin obtained from four adult female patients with clinically and histologically confirmed FFA and four age-matched female control subjects without inflammatory or scarring alopecia. Participants ranged in age from 54 to 76 years (Table 1). The primary objective was to resolve lineage- and state-specific transcriptional programs at single-cell resolution rather than global changes in cell abundance. Participants were allocated to the FFA or control group according to their clinical and histopathological diagnosis. This was an observational study, and no randomization was performed.

**Table 1:** Clinical and demographic characteristics of study participants

Sample ID	Sex	Age (years)	Group	Clinical diagnosis	FFA treatment at biopsy	Height (cm)	Weight (kg)	Relevant clinical notes
FFA005	female	76	FFA	frontal fibrosing alopecia	none	156	79	—
FFA007	female	69	FFA	frontal fibrosing alopecia	none	150	65	—
FFA010	female	65	FFA	frontal fibrosing alopecia	none	167	78	—
FFA011	female	54	FFA	frontal fibrosing alopecia	none	165	62	family history of alopecia areata
CON256	female	76	control	healthy control	N/A	N/A	N/A	—
CON265	female	63	control	healthy control (history of basal cell carcinoma)	N/A	N/A	N/A	—
CON266	female	66	control	healthy control (history of basal cell carcinoma)	N/A	N/A	N/A	latex allergy
CON267	female	75	control	healthy control (history of basal cell carcinoma)	N/A	N/A	N/A	—

The table summarizes sex, age, clinical diagnosis, treatment status at the time of biopsy, anthropometric data, and relevant clinical notes. All FFA samples were obtained from untreated patients. Control samples correspond to clinically healthy scalp tissue obtained from individuals without inflammatory scalp disease. N/A, not applicable; none, no FFA treatment at the time of biopsy; **—**, no relevant clinical notes reported.

Biopsies were obtained from clinically active frontal scalp regions exhibiting perifollicular erythema and scaling while avoiding areas of complete follicular loss or advanced fibrosis. Control samples were collected from anatomically matched frontal scalp regions to minimize variability related to scalp location and follicular density. FFA diagnosis was established by expert dermatologists based on accepted clinical and histopathological criteria.

Human clinical studies were approved by the Institutional Review Board of Reina Sofía University Hospital (Córdoba, Spain; approval ID JAAK23). All procedures complied with the Declaration of Helsinki, and written informed consent was obtained from all participants before sample collection.

Because FFA predominantly affects women, only female participants were included. Consequently, sex-specific analyses could not be performed, representing a limitation to the generalizability of the findings. All participants were adult women of White Caucasian ethnicity recruited at Reina Sofía University Hospital (Córdoba, Spain). No established cell lines or primary cell cultures were used in this study.

### Method details

#### Tissue processing and single-cell suspension generation

Fresh scalp biopsies were processed immediately after excision. Tissue was mechanically minced and enzymatically dissociated using Liberase TM (0.2%; ref. 5401119001, Roche) and Trypsin–EDTA (0.25%; ref. 25200056, Gibco). Samples were incubated at 37 °C for 30 min with gentle agitation to preserve epithelial, stromal, endothelial, and immune populations.

Following digestion, cell suspensions were filtered sequentially through 70 μm and 40 μm nylon cell strainers (ref. 352350 and 352340, Falcon) to remove debris and cell aggregates. Erythrocytes were removed by hypotonic lysis, and viable cells were enriched by density-gradient centrifugation.

Cell viability and concentration were assessed using a Countess 3 FL automated cell counter (Thermo Fisher Scientific). Preliminary optimisation experiments in human scalp samples indicated that the majority of viable cells were below 40 μm in size, and that larger aggregates were enriched in debris and low-quality events. Therefore, a final filtration step using a 40 μm strainer was applied to optimise single-cell suspension quality and minimise debris and doublets; we acknowledge that this approach may introduce bias against larger cells.

#### Droplet-based single-cell RNA sequencing

Single-cell libraries were generated using the Chromium Single Cell platform (10× Genomics) following the manufacturer’s protocols with Chromium Next GEM Single Cell 3′ v3.1 Dual Index chemistry and Next GEM Chip G. Cell encapsulation, barcoding, reverse transcription, cDNA amplification, library preparation, quality control, and sequencing were performed at the Genomics High Throughput Sequencing Facility at the University of California, Irvine.

Libraries were purified using SPRIselect beads and assessed using Bioanalyzer or TapeStation instruments prior to sequencing. Sequencing was performed on an Illumina platform to generate paired-end reads. Sequencing reads were aligned to the human reference genome (GRCh38), and gene–cell count matrices were generated using Cell Ranger v3.1.0 (10× Genomics).

#### Quality control and filtering

Quality control was performed independently for each sample. Cells were retained if they expressed >200 and <6,000 genes and exhibited <15% mitochondrial transcript content. Genes detected in fewer than three cells were excluded. Data were normalized using global-scaling normalization with a scale factor of 10,000. Low-quality clusters identified after integration, characterized by reduced gene complexity and UMI counts, were excluded from downstream analyses. Sequencing and cell recovery metrics are provided in Table S1, and pre- and post-filtering quality metrics are shown in Figure S1.

#### Data normalization, integration, and clustering

Filtered datasets were normalized using SCTransform and integrated using Seurat’s anchor-based integration workflow. Principal component analysis was performed on scaled data, and the first 20 principal components were used to construct shared nearest-neighbor graphs. Clustering was performed using modularity optimization. UMAP was used for two-dimensional visualization.

Integration quality and sample mixing were assessed by visual inspection of UMAP embeddings colored by sample identity and condition (Figures S2 and S3).

#### Cell type annotation and compartment-specific reanalysis

Cell types were annotated based on canonical marker gene expression, distinguishing epithelial, fibroblast, immune, endothelial, pericytic, adipocytic, and sebocytic compartments. Epithelial and fibroblast populations were subsequently isolated and reanalyzed independently to resolve disease-associated transcriptional states. Detailed cell-type annotations, marker genes, and cell counts are provided in Table S2.

#### Differential expression and pathway activity analysis

Differential gene expression between matched cell populations from FFA and control samples was assessed using Wilcoxon rank-sum tests with false discovery rate correction. Functional pathway activity was quantified at single-cell resolution using the UCell package, enabling rank-based enrichment scoring of curated gene signatures across cell states and pseudotime trajectories.

#### Pseudotime trajectory inference

Pseudotime trajectories were inferred separately for epithelial and fibroblast compartments using Monocle3. Root states were defined based on transcriptional proximity to control-enriched basal epithelial populations. Cells were ordered along continuous trajectories to model gradual disease-associated state transitions.

#### Cell–cell communication analysis

Intercellular communication was inferred using the CellChat R package. Ligand–receptor interactions were modeled using a mass action–based probabilistic framework. Corrections for population size were applied to minimize bias in signaling probability estimation.

#### Gene module scoring and transcription factor activity inference

Gene module activity was scored at single-cell resolution using the UCell R package. Transcription factor activity was inferred using the decoupleR framework with DoRothEA regulons. Regulon activity was analyzed across compartments, conditions, and pseudotime trajectories.

#### Histological and immunohistochemical validation

Orthogonal validation was performed using immunohistochemistry and histochemical staining on independent control and FFA scalp tissue sections.

Formalin-fixed paraffin-embedded scalp sections from control and FFA samples were stained for CK15, CXCL12/SDF1, and α-SMA. Masson trichrome staining was performed to assess perifollicular collagen deposition and fibrosis. Positive staining areas were quantified in perifollicular regions of interest and compared between groups using non-parametric testing. The antibodies and stains used are summarized in the following table.

### Quantification and statistical analysis

All analyses were performed in R (version 4.3.1). Differential gene expression and group comparisons were assessed using two-sided Wilcoxon rank-sum tests. Where applicable, *p* values were adjusted for multiple testing using the Benjamini–Hochberg false discovery rate (FDR) method, and an adjusted *p* < 0.01 was considered statistically significant. Differences in cell population proportions were evaluated using permutation-based tests. Unless otherwise indicated, *n* represents independent human biological samples (patients). For single-cell analyses, each point represents one cell, whereas statistical inference was performed at the patient level whenever applicable to minimize pseudoreplication. Data are presented as mean ± SD or median (IQR), as indicated in the corresponding figures and figure legends. Statistical significance is denoted as follows: *p* < 0.05 (∗*), p* < *0.01 (∗∗), p* < *0.001 (*∗∗∗), and *p* < 0.0001 (∗∗∗∗). Exact sample sizes (*n*), statistical tests, adjusted *p* values, definitions of error bars, and software used for each analysis are provided in the corresponding figure legends or results section. Additional analytical parameters are provided in Table S1.

## References

[1] Camacho Martínez F., García-Hernández M.J., Mazuecos Blanca J. (1999). Postmenopausal frontal fibrosing alopecia. Br. J. Dermatol..

[2] Mirmirani P., Tosti A., Goldberg L., Whiting D., Sotoodian B. (2019). Frontal Fibrosing Alopecia: An Emerging Epidemic. Skin Appendage Disord..

[3] Ko E.A., Cappetta M.E., Rangel M., Echeverria M., Abed M., Navarro Tuculet C., Mazzuoccolo L.D., Hair Care Habits, Fibrosing F. (2025). Alopecia: A Case-Control Study. Skin Appendage Disord.

[4] Oxenham A., Stevenson A. (2025). Familial Frontal Fibrosing Alopecia Occurs Early in Daughters With Affected Mothers: A Case Report and a Review of the Literature. Australas. J. Dermatol..

[5] Abdel Azim S., Bainvoll L., Vecerek N., DeLeo V.A., Adler B.L. (2025). Sunscreens part 2: Regulation and safety. J. Am. Acad. Dermatol..

[6] Rayinda T., Dand N., McSweeney S.M., Christou E., Ung C.Y., Stefanato C.M., Fenton D.A., Harries M., Palamaras I., Tidman A. (2025). Epistasis of ERAP1 With 4 Major Histocompatibility Complex Class I Alleles in Frontal Fibrosing Alopecia: A Genome-Wide Association Study Meta-Analysis. JAMA Dermatol..

[7] Shah R.R., Larrondo J., McMichael A. (2024). Is Frontal Fibrosing Alopecia Connected to Sunscreen Usage?. Cutis.

[8] Messenger A.G., Asfour L., Harries M. (2025). Frontal Fibrosing Alopecia: An Update. Am. J. Clin. Dermatol..

[9] Rayinda T., McSweeney S.M., Christou E., Ung C.Y., Fenton D.A., McGrath J.A., Dand N., Simpson M.A., Tziotzios C. (2024). Gene-Environment Interaction Between CYP1B1 and Oral Contraception on Frontal Fibrosing Alopecia. JAMA Dermatol..

[10] Kam O., Na S., Guo W., Tejeda C.I., Kaufmann T. (2023). Frontal fibrosing alopecia and personal care product use: a systematic review and meta-analysis. Arch. Dermatol. Res..

[11] Saceda-Corralo D., Ortega-Quijano D., Muñoz-Martín G., Moreno-Arrones Ó.M., Pindado-Ortega C., Rayinda T., Melián-Olivera A., Azcárraga-Llobet C., Burgos-Blasco P., Castañeda-Bermúdez M.E. (2023). Genotyping of the rs1800440 Polymorphism in CYP1B1 Gene and the rs9258883 Polymorphism in HLA-B Gene in a Spanish Cohort of 223 Patients with Frontal Fibrosing Alopecia. Acta Derm. Venereol..

[12] Porriño-Bustamante M.L., Montero-Vílchez T., Pinedo-Moraleda F.J., Fernández-Flores Á F.-P.M.A., Arias-Santiago S., Frontal Fibrosing Alopecia, Use S. (2022). A Cross-sectional Study of Actinic Damage. Acta Derm. Venereol..

[13] Aldoori N., Dobson K., Holden C.R., McDonagh A.J., Harries M., Messenger A.G. (2016). Frontal fibrosing alopecia: possible association with leave-on facial skin care products and sunscreens; a questionnaire study. Br. J. Dermatol..

[14] Harries M.J., Meyer K., Chaudhry I., E Kloepper J., Poblet E., Griffiths C.E., Paus R. (2013). E Kloepper J, Poblet E, Griffiths CE, Paus R. Lichen planopilaris is characterized by immune privilege collapse of the hair follicle's epithelial stem cell niche. J. Pathol..

[15] Moreno-Arrones O.M., Saceda-Corralo D., Fonda-Pascual P., Rodrigues-Barata A.R., Buendía-Castaño D., Alegre-Sánchez A., Pindado-Ortega C., Molins M., Perosanz D., Segurado-Miravalles G. (2017). Frontal fibrosing alopecia: clinical and prognostic classification. J. Eur. Acad. Dermatol. Venereol..

[16] Saceda-Corralo D., Pindado-Ortega C., Moreno-Arrones O.M., Ortega-Quijano D., Fernández-Nieto D., Jiménez-Cauhe J., Vañó-Galván S. (2020). Association of Inflammation With Progression of Hair Loss in Women With Frontal Fibrosing Alopecia. JAMA Dermatol..

[17] Del Duca E., Ruano Ruiz J., Pavel A.B., Sanyal R.D., Song T., Gay-Mimbrera J., Zhang N., Estrada Y.D., Peng X., Renert-Yuval Y. (2020). Frontal fibrosing alopecia shows robust T helper 1 and Janus kinase 3 skewing. Br. J. Dermatol..

[18] Wang E.H.C., Monga I., Sallee B.N., Chen J.C., Abdelaziz A.R., Perez-Lorenzo R., Bordone L.A., Christiano A.M. (2022). Primary cicatricial alopecias are characterized by dysregulation of shared gene expression pathways. PNAS Nexus.

[19] Rivera-Ruiz I., Ungar B., Dávila-Flores V., Gay-Mimbrera J., Gómez-Arias P.J., Juan-Cencerrado M., Mochón-Jiménez C., Parra-Peralbo E., Isla-Tejera B., López-Viñau López T. (2025). Unravelling the transcriptomic landscape of primary lymphocytic scarring alopecias: systematic review and meta-analysis. Front. Immunol..

[20] Imanishi H., Ansell D.M., Chéret J., Harries M., Bertolini M., Sepp N., Bíró T., Poblet E., Jimenez F., Hardman J. (2018). Epithelial-to-Mesenchymal Stem Cell Transition in a Human Organ: Lessons from Lichen Planopilaris. J. Invest. Dermatol..

[21] Mitamura Y., Reiger M., Kim J., Xiao Y., Zhakparov D., Tan G., Rückert B., Rinaldi A.O., Baerenfaller K., Akdis M. (2023). Spatial transcriptomics combined with single-cell RNA-sequencing unravels the complex inflammatory cell network in atopic dermatitis. Allergy.

[22] Jiang R., Fang Z., Tsoi L.C., Gudjonsson J.E. (2026). Inflammatory niches as spatial drivers of disease mechanisms and targets for personalized treatment. J. Eur. Acad. Dermatol. Venereol..

[23] Leung A., Marquez-Grap G., Kranyak A., Liao W. (2025). A review of spatial transcriptomics in psoriasis: new insights into cellular contributions. Curr. Opin. Immunol..

[24] Houser A.E., Kazmi A., Nair A.K., Ji A.L. (2023). The Use of Single-Cell RNA-Sequencing and Spatial Transcriptomics in Understanding the Pathogenesis and Treatment of Skin Diseases. JID Innov..

[25] Kumaran G., Carroll L., Muirhead N., Bottomley M.J. (2025). How Can Spatial Transcriptomic Profiling Advance Our Understanding of Skin Diseases?. J. Invest. Dermatol..

[26] Yao W.H., Sun C., Su Z., Wang P., Zeng Y.P. (2025). The Role of Fibroblasts in Atopic Dermatitis: Establishing Proinflammatory Microenvironments and Mediating Cellular Crosstalk. J. Inflamm. Res..

[27] Chen Q., Li Y., Zhu Q., Li Z., Shao G., Liu Y., Jiang P., Tao Q., Shen L., Zhu J. (2025). Single-cell sequencing combined with spatial transcriptomics reveals the characteristics of follicle-targeted inflammation patterns in primary cicatricial alopecia. Cell Biosci..

[28] Yokota J., Gomi T., Harada Y., Yo K., Ahn K., Sanzen N., Machida H., Shirai Y., Fujiwara H. (2025). Single-cell transcriptomic reconstruction of the human hair cycle: Capturing the temporal dynamics of skin tissue remodeling. Cell Rep..

[29] Ito T. (2010). Hair follicle is a target of stress hormone and autoimmune reactions. J. Dermatol. Sci..

[30] Klimitz F.J., Shen Y., Repetto F., Brown S., Knoedler L., Ko C.J., Abu Hussein N., Crisler W.J., Adams T., Kaminski N. (2025). Keratinocytes as active regulators of cutaneous and mucosal immunity: a systematic review across inflammatory epithelial disorders. Front. Immunol..

[31] Restrepo P., Wilder A., Houser A., Sandhu H.S., Ramirez A., Grace Hren M., Gill R., Kazmi A., Chen L., Nigro A. (2026). Single-cell spatial transcriptomic analysis of human skin anatomy. Nat. Genet..

[32] Tissot N., Genty G., Santoprete R., Baltenneck F., Thibaut S., Michelet J.F., Sequeira I., Bornschlögl T. (2025). Mapping cell dynamics in human ex vivo hair follicles suggests pulling mechanism of hair growth. Nat. Commun..

[33] Andersen B., Duan J., Karri S.S. (2023). How and Why the Circadian Clock Regulates Proliferation of Adult Epithelial Stem Cells. Stem Cell..

[34] Greenberg E.N., Marshall M.E., Jin S., Venkatesh S., Dragan M., Tsoi L.C., Gudjonsson J.E., Nie Q., Takahashi J.S., Andersen B. (2020). Circadian control of interferon-sensitive gene expression in murine skin. Proc. Natl. Acad. Sci. USA.

[35] Deshayes N., Genty G., Berthelot F., Paris M. (2018). Human long-term deregulated circadian rhythm alters regenerative properties of skin and hair precursor cells. Eur. J. Dermatol..

[36] Al-Nuaimi Y., Hardman J.A., Bíró T., Haslam I.S., Philpott M.P., Tóth B.I., Farjo N., Farjo B., Baier G., Watson R.E.B. (2014). A meeting of two chronobiological systems: circadian proteins Period1 and BMAL1 modulate the human hair cycle clock. J. Invest. Dermatol..

[37] Duan J., Greenberg E.N., Karri S.S., Andersen B. (2021). The circadian clock and diseases of the skin. FEBS Lett..

[38] Yu G.T., Ganier C., Allison D.B., Tchkonia T., Khosla S., Kirkland J.L., Lynch M.D., Wyles S.P. (2025). Mapping epidermal and dermal cellular senescence in human skin aging. Aging Cell.

[39] Steele L., Olabi B., Roberts K., Mazin P.V., Koplev S., Tudor C., Rumney B., Admane C., Jiang T., Correa-Gallegos D. (2025). A single-cell and spatial genomics atlas of human skin fibroblasts reveals shared disease-related fibroblast subtypes across tissues. Nat. Immunol..

[40] Yang Q., Zhang J., Bao Q., Zhong J., Wang X., Tao Y., Xu X., Lv K., Wang Y., Li B. (2022). Foxp1 and Foxp4 Deletion Causes the Loss of Follicle Stem Cell Niche and Cyclic Hair Shedding by Inducing Inner Bulge Cell Apoptosis. Stem Cell..

[41] Marella S., Plikus M., Gudjonsson J.E. (2026). Skin fibroblasts in health and disease: From extracellular matrix remodeling to immune regulation. J Invest Dermatol.

[42] Almet A.A., Liu Y., Nie Q., Plikus M.V., Integrated Single-Cell Analysis Reveals Spatially, Dynamic T. (2025). Heterogeneity in Fibroblast States during Wound Healing. J. Invest. Dermatol..

[43] Abouhashem A.S., Saber S.K., Abouzekry S., Elkholy M., Moustafa A., Abdellatif A., Azzazy H.M.E., Elbaz A.A., Singh K., Sen C.K., Sharara H. (2025). Identification of Skin Multicellular Reprogramming Factors as Potential Treatment for Nonhealing Diabetic Foot Ulcers. Adv. Wound Care.

[44] Mochón-Jiménez C., Rivera-Ruiz I., Gómez-Arias P.J., de Luque-Fernández J., Gay-Mimbrera J., Ruano J. (2026). Disease-dependent accessibility of hair follicle compartments revealed by tape-strip transcriptomics. Br J Dermatol.

[45] Mochón-Jiménez C., Gay-Mimbrera J., Dávila-Flores V., He H., Zhou J., Rivera-Ruiz I., Gómez-Arias P.J., Luque-Fernández J., Ungar B., Guttman-Yassky E., Ruano J. (2026). Non-Invasive Scalp Tape-Strip RNA Sequencing Captures Disease Activity and Treatment-Response Signatures in Alopecia Areata. Allergy.

[46] Qiu X., Mao Q., Tang Y., Wang L., Chawla R., Pliner H.A., Trapnell C. (2017). Reversed graph embedding resolves complex single-cell trajectories. Nat. Methods.

[47] Trapnell C., Cacchiarelli D., Grimsby J., Pokharel P., Li S., Morse M., Lennon N.J., Livak K.J., Mikkelsen T.S., Rinn J.L. (2014). The dynamics and regulators of cell fate decisions are revealed by pseudotemporal ordering of single cells. Nat. Biotechnol..

[48] Aibar S., González-Blas C.B., Moerman T., Huynh-Thu V.A., Imrichova H., Hulselmans G., Rambow F., Marine J.C., Geurts P., Aerts J. (2017). single-cell regulatory network inference and clustering. Nat. Methods.

[49] Garcia-Alonso L., Holland C.H., Ibrahim M.M., Turei D., Saez-Rodriguez J. (2019). Benchmark and integration of resources for the estimation of human transcription factor activities. Genome Res..

