# Supplementary material for: Single-cell transcriptomics reveals epithelial-stromal crosstalk underlying fibrotic remodeling in frontal fibrosing alopecia
Source: iScience. 2026 Jul 18;29(8):116854. doi: 10.1016/j.isci.2026.116854 (PMC13400765; doi:10.1016/j.isci.2026.116854)
Supplement: Document S1. Tables S1–S3 and Figures S1–S8 [file mmc1.pdf]

iScience, Volume *29*

Supplemental information

Single-cell transcriptomics reveals epithelial-stromal crosstalk underlying fibrotic remodeling in frontal fibrosing alopecia

Viviana Dávila-Flores, Jesús Gay-Mimbrera, Carmen Mochón-Jiménez, Irene Rivera-Ruiz, Juan de Luque-Fernández, Pedro J. Gómez-Arias, Beatriz Isla-Tejera, Benjamin Ungar, Benjamin D. Hu, Helen He, Fernando Leiva-Cepas, Emma Guttman-Yassky, and Juan Ruano

SUPPLEMENTARY FIGURES

Figure S1. Pre-filtering and post-filtering quality control metrics for scRNA-seq samples. Violin and box plots showing distributions of nFeature_RNA, nCount_RNA and percent mitochondrial reads (percent.mt) before (left) and after (right) quality filtering, aggregated by group (FFA vs control). Pre-filtering distributions are shown in grey and post-filtering distributions in blue.

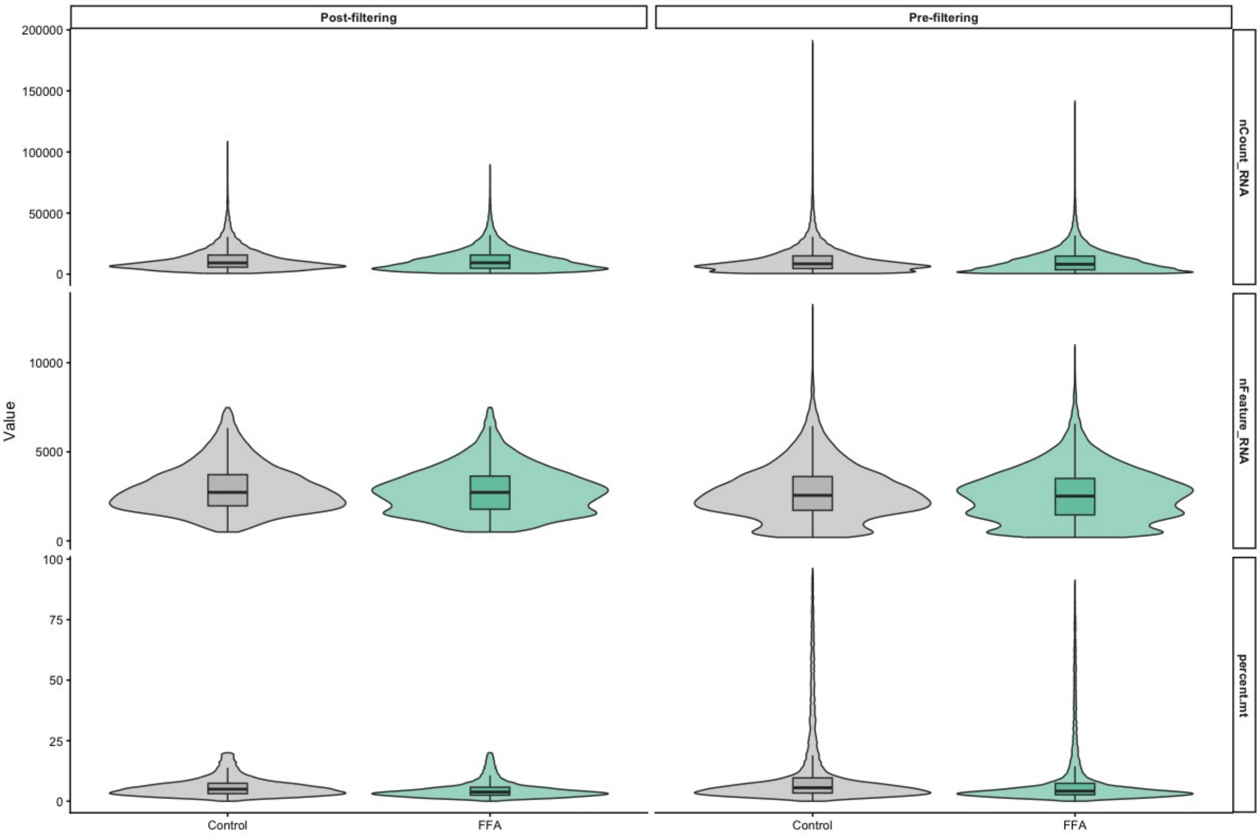

Figure S2. Global cellular landscape of human scalp skin revealed by single-cell RNA sequencing. Global UMAP representation of the integrated single-cell RNA-sequencing dataset, coloured by major cellular compartments of human scalp skin, including epithelial, fibroblast, myofibroblast, immune, endothelial, pericyte/smooth muscle, adipocyte and sebocyte populations. Cells not meeting stringent criteria for confident lineage assignment are shown as Unassigned. This global overview confirms preservation of the overall cellular architecture across samples and served as the reference framework for subsequent compartment-specific analyses focusing on epithelial and fibroblast populations.

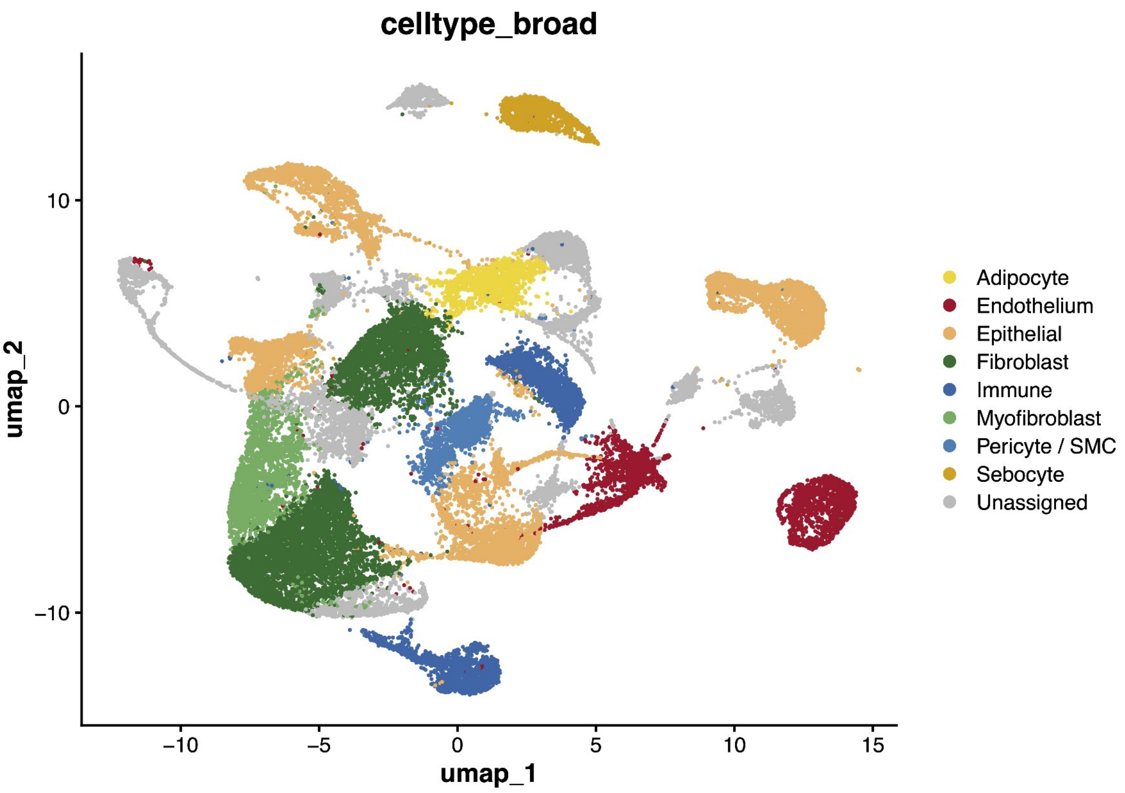

Figure S3. Global UMAP coloured by sample and condition. (A) UMAP of the integrated dataset coloured by sample ID, showing broad inter-sample mixing across the embedding. (B) The same UMAP coloured by condition (FFA vs control), illustrating substantial global overlap between conditions, consistent with preserved overall cellular architecture at the whole-tissue level.

A

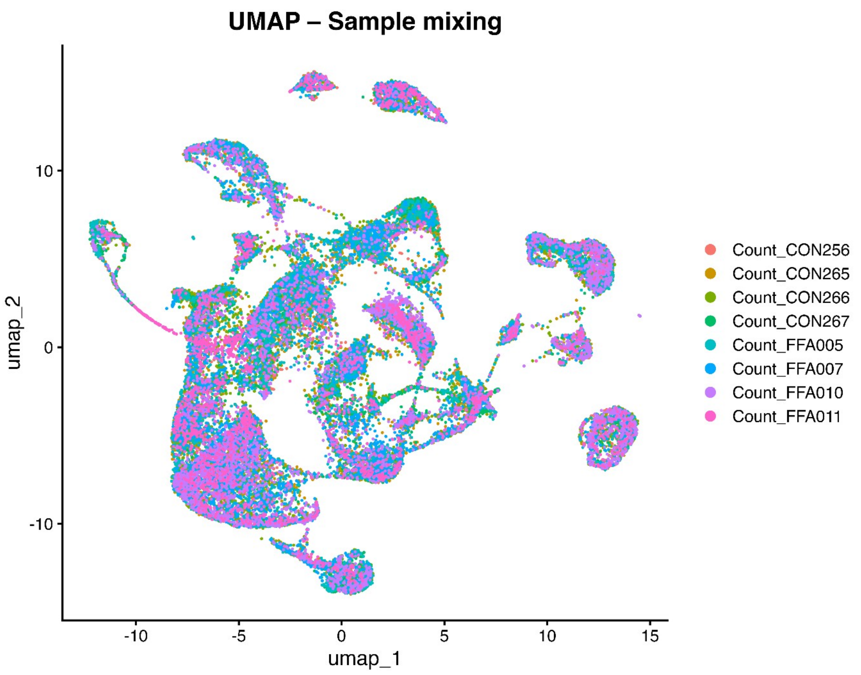

B

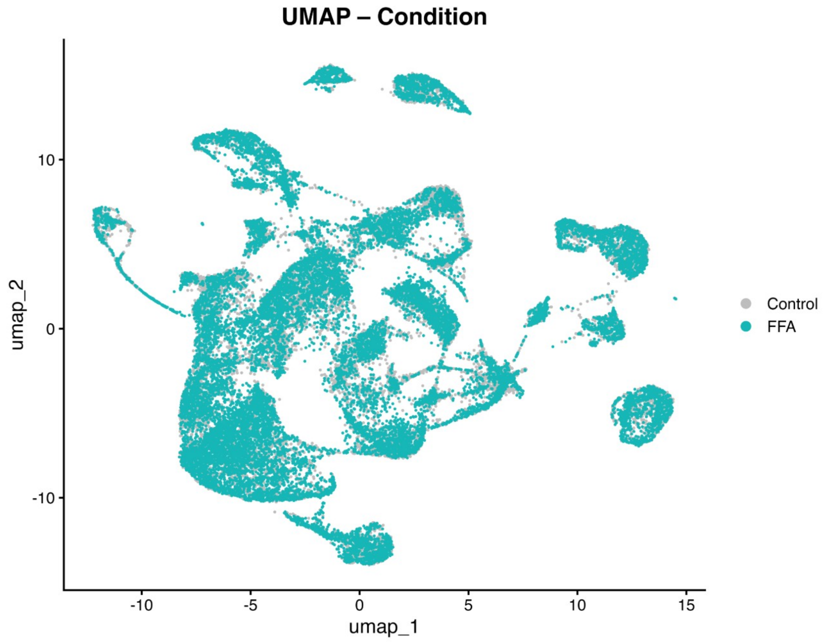

Figure S4. Spatial distribution of canonical lineage markers on the integrated UMAP. Feature plots showing the expression of representative marker genes projected onto the integrated UMAP embedding. Markers highlight major scalp cell lineages, including immune cells (PTPRC, NKG7, LYZ), fibroblast and myofibroblast populations (COL1A1, DCN, ACTA2), vascular and lymphatic endothelium (PECAM1, PROX1), and epithelial compartments (KRT14, KRT15, SOX9). Colour intensity reflects normalized gene expression levels.Broad distribution of canonical epithelial markers (KRT14, KRT15) reflects biological overlap between closely related epithelial states and potential technical effects (e.g., ambient RNA, low-level doublets). The spatial segregation of these markers corroborates the marker-based cluster annotation and confirms preservation of expected lineage architecture within the integrated dataset.

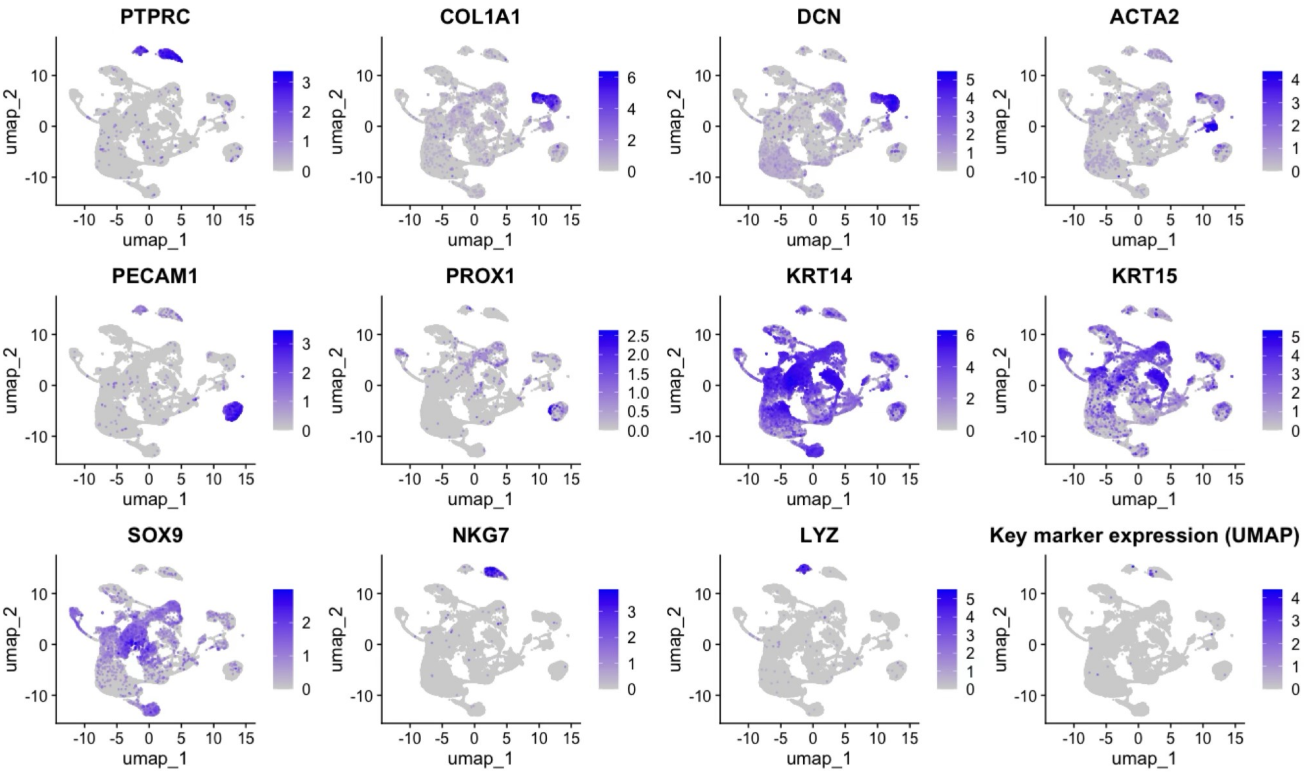

Figure S5. Sample-level broad cell-type composition across control and FFA samples.
Bubble heatmap showing the relative abundance of broad cellular compartments per sample. Bubble size and color both indicate within-sample proportion (%). Samples are stratified by condition (Control, FFA). Major compartments, including epithelial, fibroblast, and immune populations, are represented across all samples, including lower-cell-count controls (CON266 and CON267), although low-frequency populations show expected variability with sampling depth.

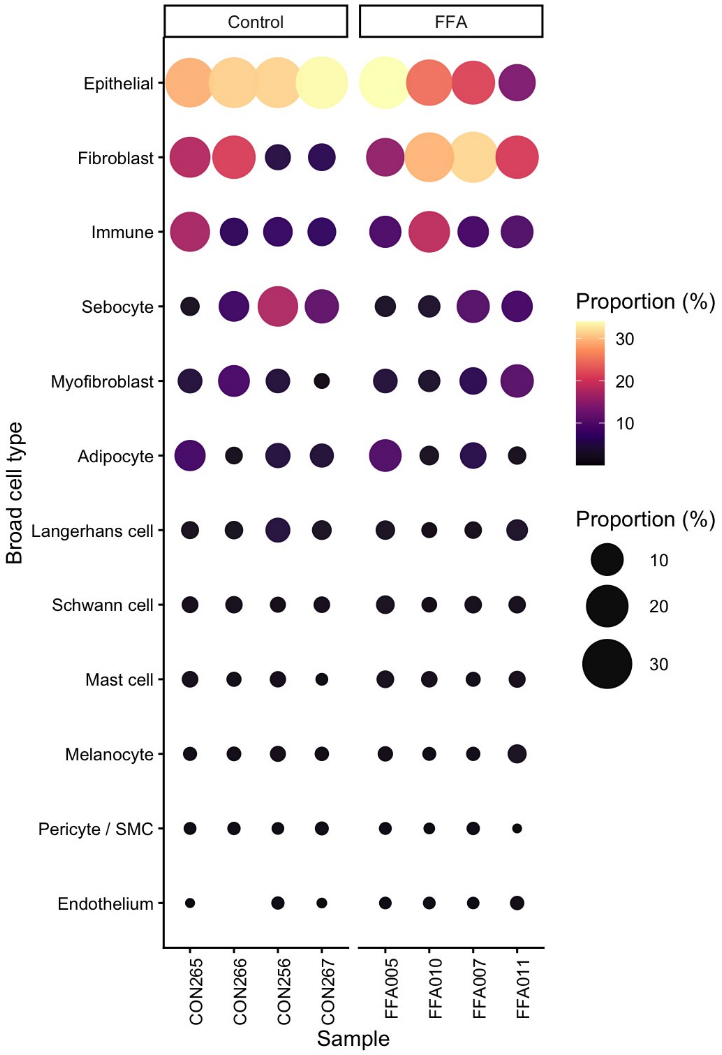

Figure S6. EMT-related signal across epithelial states in FFA and control. Top: Dot plot of canonical EMT markers across epithelial states (IF_basale, ORS_basal, ORS_suprabasal, IRS, HairMatrix_precortical, HairMatrix_germinal, Stress_response). Dot size indicates percent expressed cells and color indicates average scaled expression. Core EMT transcription factors (SNAI1, SNAI2, ZEB1, ZEB2, TWIST1) show low and heterogeneous expression across states. Middle: Hallmark EMT module score by group (Control vs FFA), showing no strong global shift. Bottom: Hallmark EMT module score across epithelial states, indicating state-dependent variation.

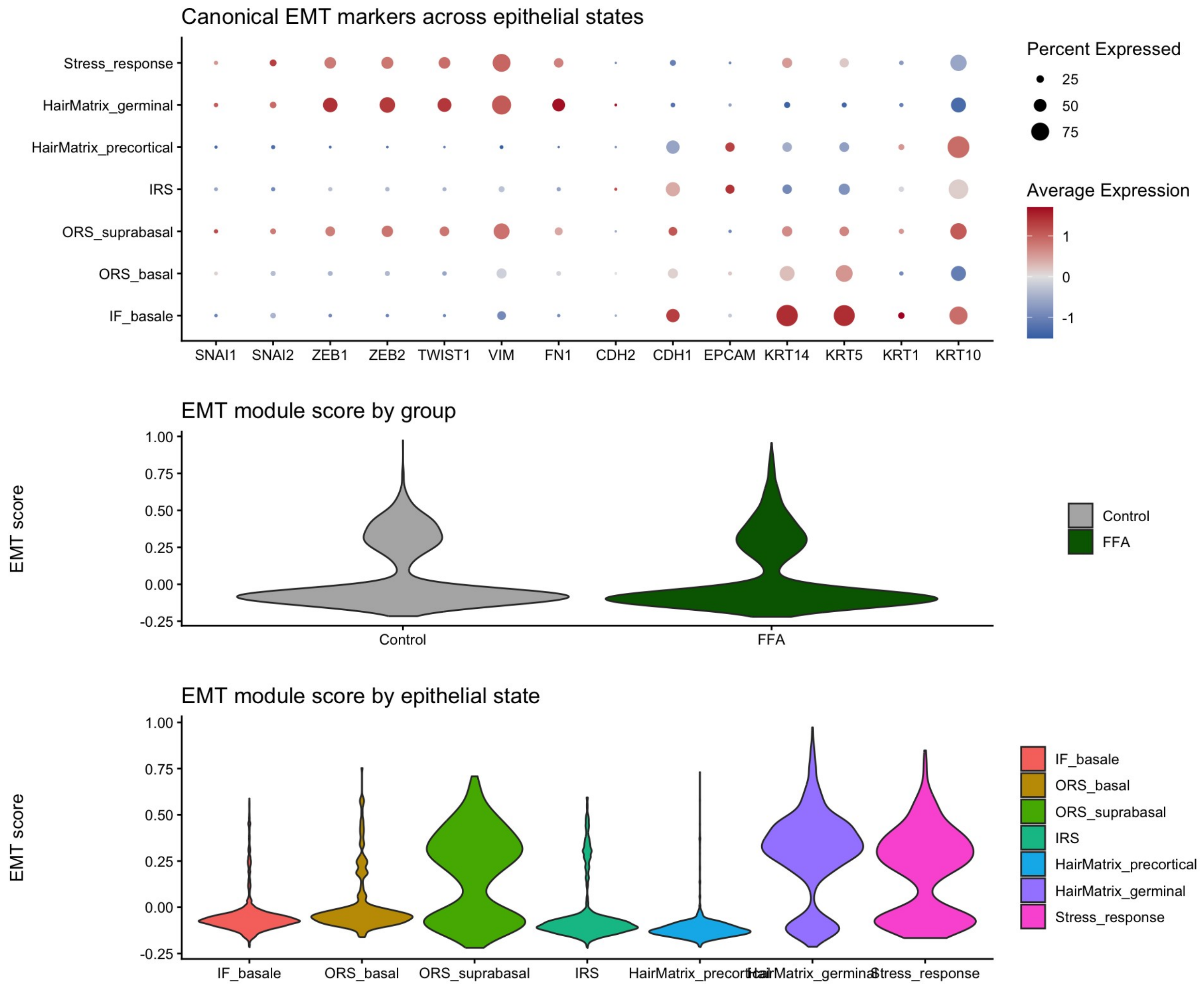

Figure S7. State-specific comparison of EMT module score between Control and FFA. Violin/box plots of epithelial EMT module score stratified by epithelial state and group, with per-state significance annotation. Statistical testing was performed using two-sided Wilcoxon rank-sum tests with BH correction across states. Significance labels: ns, not significant; *P < 0.05; **P < 0.01; ***P < 0.001.

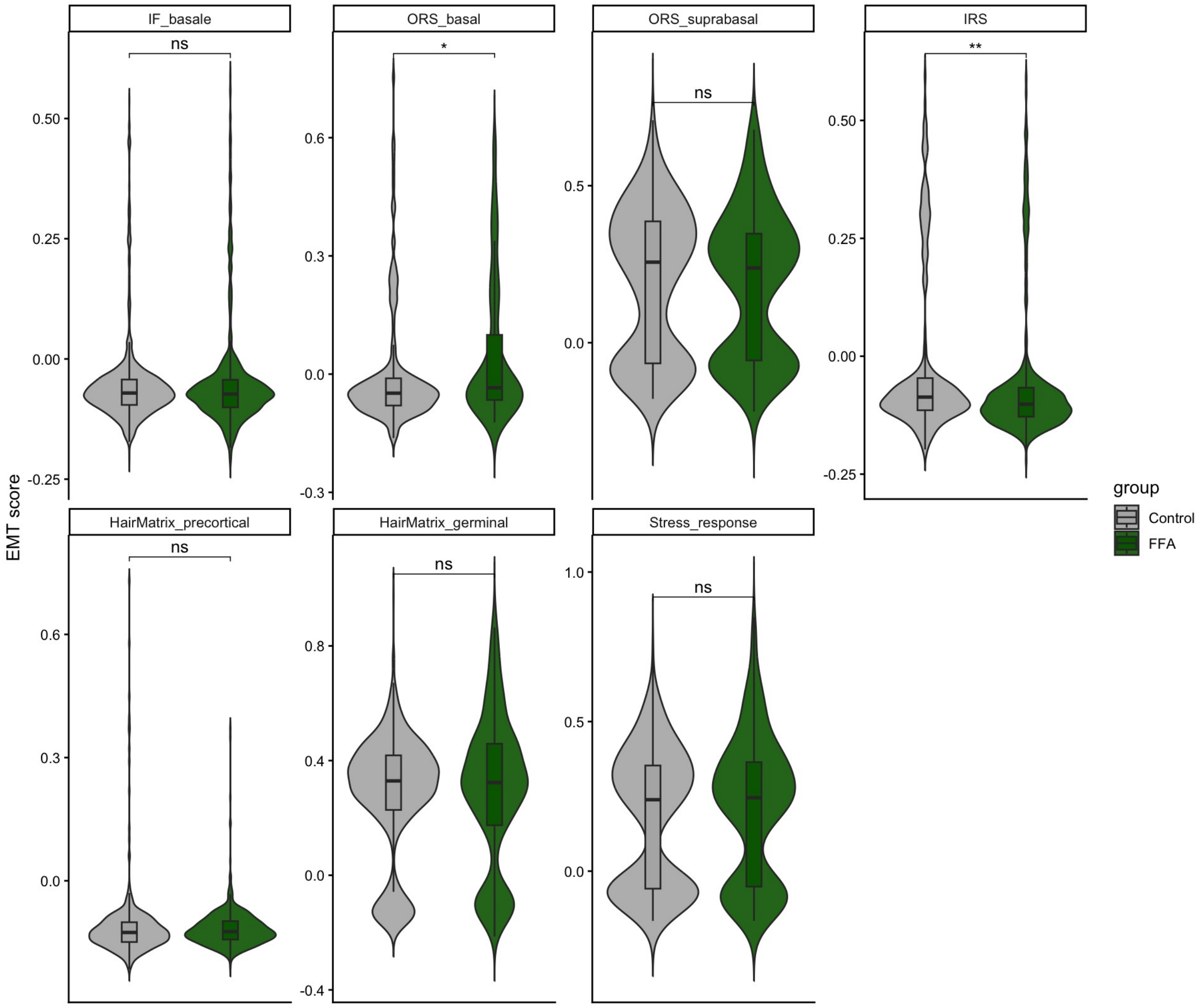

Figure S8. Orthogonal histopathological support for epithelial niche alteration, stromal activation and perifollicular fibrosis in frontal fibrosing alopecia (FFA). Representative immunohistochemical staining of CK15, SDF1/CXCL12 and α-SMA, together with Masson trichrome staining, in control and FFA scalp tissue sections. FFA samples showed reduced CK15 staining within bulge-associated epithelial regions, increased perifollicular SDF1/CXCL12 and α-SMA expression, and enhanced perifollicular collagen deposition compared with control scalp. Quantitative analyses of perifollicular positive-stained area are shown on the right. Scale bars: 100 µm.

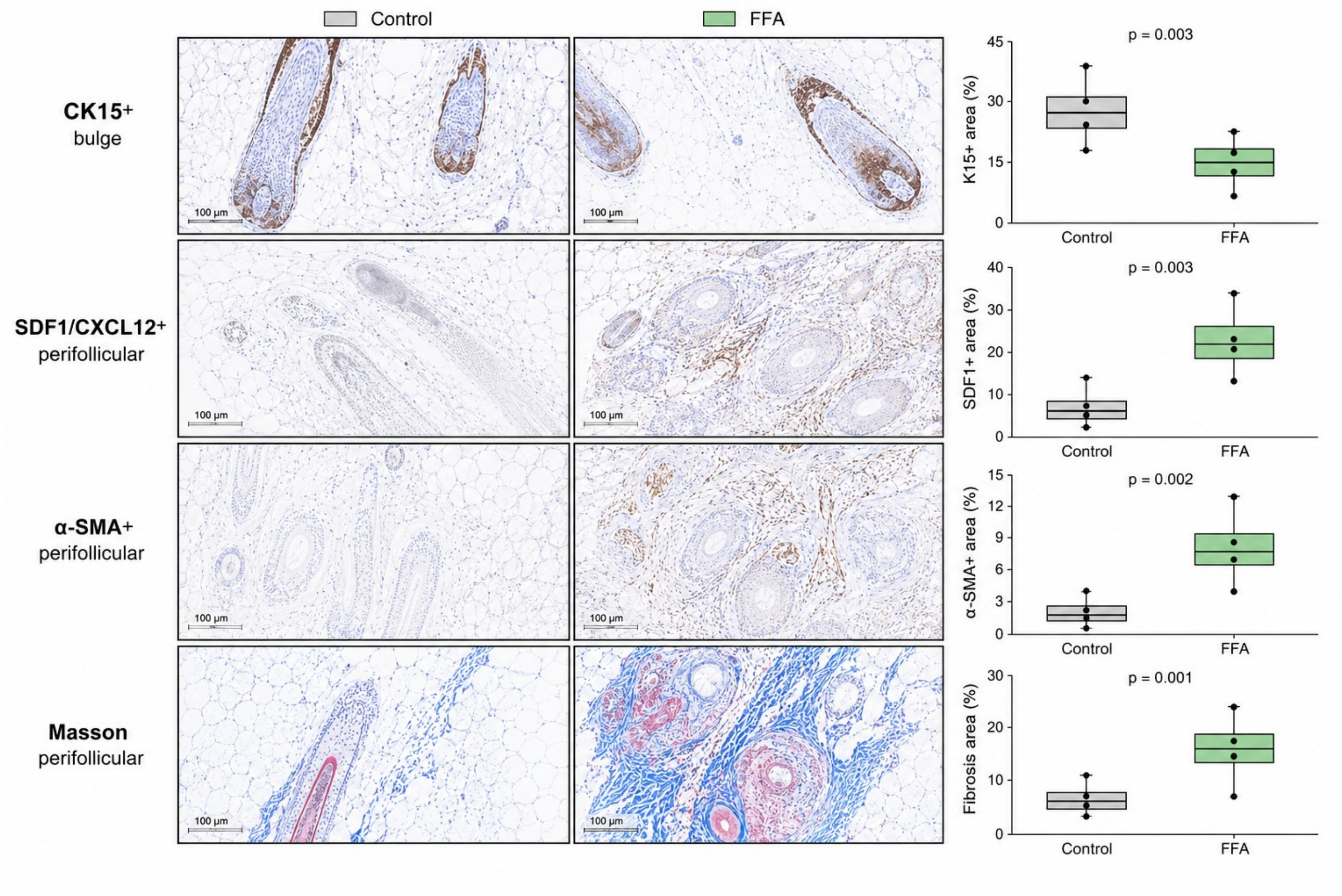

SUPPLEMENTARY TABLES

Table S1. Single-cell RNA sequencing quality metrics. Values are reported as approximate ranges derived from Cell Ranger output summaries. All samples met standard quality thresholds for downstream single-cell analyses.

Sample ID	Condition	Cells recovered (approx.)	Mean genes per cell (approx.)
FFA005	FFA	~7,500	~2,800
FFA007	FFA	~6,800	~2,600
FFA010	FFA	~5,200	~2,100
FFA011	FFA	~4,900	~2,000
CON256	Control	~8,900	~2,900
CON265	Control	~6,200	~2,300
CON266	Control	~3,100	~1,800
CON267	Control	~2,600	~1,700

Table S2. Cell-type annotation and representative marker genes. Cell-type annotation of the integrated scalp single-cell RNA-sequencing dataset. Cells were assigned to major tissue compartments and specific cell types based on cluster-level differential expression analysis and curated, literature-supported marker gene panels. For each population, the table reports the major lineage, representative marker genes, and the number of cells. Cell counts correspond to the final dataset after quality control, filtering, and annotation refinement. Cells classified as *Unassigned / low-confidence* did not meet stringent marker-based criteria for confident lineage attribution and were retained to avoid forced or potentially misleading annotation.

Compartment	Cell type	Representative marker genes	Number of cells
**Epithelial**	Basal / ORS keratinocytes	KRT14, KRT5, KRT15, TP63	8,677
	Hair shaft keratinocytes	KRT31, KRT32, KRT33A, KRT85	2,041
	Hyperproliferative epithelium	KRT6A, KRT16, KRT17	2,137
	IFE / Infundibulum	KRT1, KRT10, IVL, FLG	2,409
	Inner root sheath (IRS)	KRT71, KRT72, KRT73, KRT75	2,090
**Sebaceous lineage**	Sebocytes	KRT7, KRT18, SOX9, MGST1	1,424
**Stromal**	Fibroblasts (total)	COL1A1, COL1A2, DCN, LUM	8,242
	Papillary-like fibroblasts	COL1A1, DCN, LUM, FBLN1	5,000
	Reticular-like fibroblasts	COL3A1, COL6A1, FBLN2	3,242
	ACTA2^{+} myofibroblasts	ACTA2, TAGLN, POSTN, FN1	2,470
	Adipocytes	ADIPOQ, PLIN1, FABP4	1,516
	Pericytes / smooth muscle–like cells	RGS5, PDGFRB, MCAM, CSPG4	1,518
**Vascular**	Vascular endothelium	PECAM1, VWF, KDR	1,615
	Lymphatic endothelium	PDPN, LYVE1, PROX1	1,601
**Immune**	Immune cells (total)	PTPRC, LST1	3,717
	T / NK cells	CD3D, CD3E, NKG7, GNLY	2,025

Compartment	Cell type	Representative marker genes	Number of cells
	Myeloid cells	LYZ, LST1, FCGR3A	1,692
	Langerhans cells	CD207, CD1A, FCER1A	418
	Mast cells	TPSAB1, TPSB2, KIT	216
**Neural crest–derived**	Melanocytes	PMEL, MLANA, TYR	181
	Schwann cells	SOX10, S100B, MPZ	275
**Other**	Unassigned / low-confidence clusters	–	7,124
